# Age at Autism Diagnosis in the Norwegian Mother, Father, and Child Cohort Study (MoBa): Exploring the Association With Family Well-Being

**DOI:** 10.1177/13623613261474929

**Published:** 2026-08-22

**Authors:** Bernt Damian Glaser, Meseret Mamo Bazezew, Daniela Bragantini, Laura Elizabeth Hegemann, Stian Orm, Morgan James Morgan, Somer L. Bishop, Stian Barbo Valand, Laurie John Hannigan, Alexandra Havdahl

**Affiliations:** 1 Norwegian Institute of Public Health, Oslo, Norway; 2 University of Oslo, Norway; 3 Lovisenberg Diaconal Hospital, Oslo, Norway; 4 University of Inland Norway, Lillehammer, Norway; 5 Division of Mental Health Care, Innlandet Hospital Trust, Norway; 6 University of Surrey, Guildford, UK; 7 University of California–San Francisco, USA; 8 University of Bristol, UK

**Keywords:** autism, diagnosis, well-being, family functioning and support, school-age children, adolescents, parents, MoBa, the Norwegian Mother, Father and Child Cohort Study

## Abstract

For many autistic individuals, close family members represent a crucial source of support throughout their lifetime. Early autism diagnosis is thought to benefit both autistic individuals and their family members by facilitating early access to services and information. However, little research has empirically studied the relationship between age at diagnosis and well-being among autistic individuals and their family members. In this study, we examined predictors of age at autism diagnosis and its relationship with family well-being using the population-based Norwegian Mother, Father, and Child Cohort Study (MoBa). We found that the most important factors associated with a later age at diagnosis were fewer social communication difficulties at age three, fewer maternal concerns about development in early childhood, absence of a co-occurring intellectual disability diagnosis, female sex, and having older siblings. While a later age at diagnosis was associated with higher well-being for mothers and children during childhood, it was associated with lower well-being for children during adolescence. All associations were substantially attenuated after adjustment for various clinical, demographic, and genetic factors – reflecting complex patterns of confounding. We argue that future research should aim to leverage public awareness campaigns, screening programmes, or changes in clinical practice to understand the causal mechanisms underlying these relationships.

**Lay abstract:**

Family members of autistic people are often an important source of support for their well-being. An earlier autism diagnosis might be helpful for autistic individuals and their families, because families might receive professional support sooner. However, the impact of an earlier diagnosis is difficult to study, because many child and family characteristics that lead to an earlier autism diagnosis may have their own influence on well-being in autistic individuals and their families. In this study, we look at how age at diagnosis and family well-being are related and how they both relate to different child and family characteristics. We find that children, on average, receive a later autism diagnosis when they have fewer social communication difficulties and fewer maternal concerns in early childhood, do not also have an intellectual disability diagnosis, are a girl, or have older siblings. We also found that a later autism diagnosis was associated with higher well-being for children and mothers in childhood but with lower well-being for children in adolescence. When we compared families with similar child and family characteristics, we no longer saw such clear links between age at diagnosis and well-being. This might be because of the complex mixture of influences child and family characteristics have on both age at diagnosis and well-being and suggests that other types of study may be better for investigating the impact of age at diagnosis on well-being in families.

## Background

Families are often the primary source of support for autistic individuals throughout their lives (Lord et al., 2020). The family environment can both influence and be influenced by the social, functional, behavioural, and emotional difficulties that autistic children may experience (Lord et al., 2022; Tint & Weiss, 2016; Whitehead et al., 2015). Although parents commonly report both positive and negative experiences associated with having an autistic child (Myers et al., 2009; Tint & Weiss, 2016; Whitehead et al., 2015), they generally report lower well-being than parents of children with other neurodevelopmental or psychiatric conditions or typically developing children (Tint & Weiss, 2016). Siblings of autistic children also often face unique challenges, such as getting less attention from their parents, having to sacrifice their own activities, and dealing with challenging behaviours from their autistic sibling (Myers et al., 2009; Tudor et al., 2018). Research has increasingly focused on identifying factors that may explain differences in well-being between families with and without autism, but there is still limited understanding of the factors that lead to variation in well-being within families of autistic children.

The timing of receiving an autism diagnosis is one such hypothesised factor that impacts family well-being. The diagnosis of autism plays a crucial role in providing access to support services. While autism has historically been understood as an early emerging neurodevelopmental condition, recent findings indicate there is wider variability in early development, and an increasing number of autistic people receive their diagnoses in middle childhood and later (Schendel & Thorsteinsson, 2018). Previous research, primarily in clinical and cross-sectional samples, shows that a substantial proportion of the variation in age at diagnosis can be explained by differences in clinical and demographic factors, where an earlier age at diagnosis is associated with being male, more social communication difficulties, co-occurring intellectual disability, higher household income, and higher parental education (Daniels & Mandell, 2014; Loubersac et al., 2023). Moreover, longitudinal data and prospective general population samples are needed to better understand the variability in age at diagnosis, its predictors and the relationship with family well-being.

Randomised trials of interventions designed to facilitate earlier diagnoses of autism, with follow-up of well-being among diagnosed individuals and their family members, would provide “gold-standard” evidence on this relationship. There is some evidence that early identification efforts can decrease the age at autism diagnosis (Al-Mamari et al., 2025), and there is evidence that an earlier onset of interventions for autistic children leads to greater developmental gains (Fuller & Kaiser, 2020; Towle et al., 2020), although well-being outcomes have not been assessed. In the absence of evidence from randomised controlled trials, observational data – particularly data from prospectively recruited longitudinal cohorts – can be used to investigate the relationship between age at first autism diagnosis and family well-being. However, in observational research, it is important to consider individual and family characteristics that may confound the relationship between autism diagnosis and family well-being. Many of the aforementioned factors that are associated with receiving an earlier autism diagnosis (Daniels & Mandell, 2014; Loubersac et al., 2023) are also associated with lower well-being among members of the nuclear family in the context of autism (Tint & Weiss, 2016), potentially leading to “third variable” confounding. For example, co-occurring intellectual disability may lead to earlier referral and diagnosis but may also influence the well-being of autistic children and their family members, which could induce an association between age at diagnosis and family well-being.

Considering these potential confounding mechanisms, we expect an earlier autism diagnosis to be associated with lower well-being among autistic individuals, their parents, and their siblings in observational data and a weakening or reversal of this relationship when adjusting for hypothesised clinical and sociodemographic predictors of age of diagnosis – consistent with confounding “masking” a beneficial underlying impact of earlier diagnosis on family well-being. Therefore, in this preregistered study, we evaluate this expectation by characterising the variability in age at diagnosis, its predictors, the observational relationship between age at autism diagnosis and the well-being of different members of the family, and how it changes when adjusting for potential confounders in a large prospective, population-based pregnancy cohort. Using linked questionnaire, genomic, and health-registry data from Norwegian families, we ask (i) to what extent age at autism diagnosis is associated with the well-being of autistic children, their parents, and their siblings and (ii) to what extent these associations change when accounting for individual and family characteristics that predict age at diagnosis. Finally, given that females, on average, receive an autism diagnosis later than males (Dalsgaard et al., 2020), we also assess whether the relationship between age at first diagnosis and family members’ well-being is different for families of male and female autistic children.

## Methods

The methods for this study were preregistered (Glaser et al., 2025).

### Sample

We used questionnaire, genomic, and health-registry data from the Norwegian Mother, Father, and Child Cohort Study (MoBa; Brandlistuen et al., 2025), a population-based pregnancy cohort study conducted by the Norwegian Institute of Public Health. Participants were recruited from all over Norway from 1999 to 2008. The women consented to participation in 41% of the pregnancies. The cohort includes approximately 114,500 children, 95,200 mothers, and 75,200 fathers. The MoBa is regulated by the Norwegian Health Registry Act. The current study was approved by the Regional Committees for Medical and Health Research Ethics (2016/1702).

For this study, we use a subsample of the MoBa cohort containing *N* = 2,483 children who had an autism diagnosis recorded on at least two occasions between 2008 and 2023 in the Norwegian Patient Registry (NPR). The NPR contains all diagnoses made in the specialist health care system in Norway, according to ICD-10 (Bakken et al., 2020). The clinical guidelines in Norway state that diagnostic assessments of autism are to be carried out in the specialist health care system. We also include the mothers (*N* = 2,483), fathers (*N* = 1,887), and siblings (*N* = 820) of those children diagnosed with autism.

### Measures

#### Age at First Diagnosis

Our exposure variable is age at first autism diagnosis, which is predominantly retrieved from the NPR. The NPR data are only available from 2008, meaning that the earliest possible NPR-registered diagnosis for the oldest children in MoBa, born in 1999, would be at age 9, while the youngest participants would have NPR data since birth. To account for censoring of the age at first diagnosis for older children in our sample, we implemented the following pipeline (see Figure 1; see details in Supplementary Materials).

**Figure 1.**
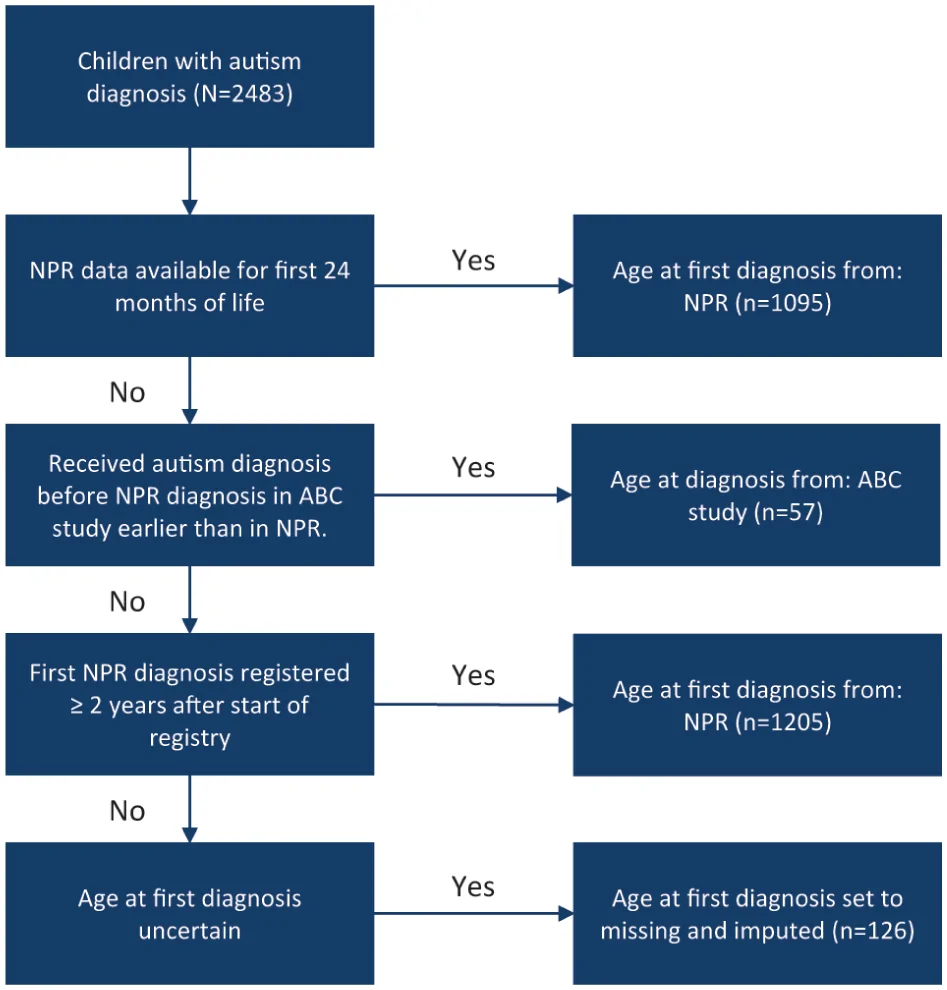
A flowchart specifying the origin of the age at first autism diagnosis variable for all autistic children.

#### Outcome Variables

The outcome of interest is the well-being of each member of the nuclear family, where well-being consists of life satisfaction, depression, and anxiety. Each component was measured during middle childhood (age 8) and adolescence (age 14) for autistic children, siblings, and mothers. Fathers rated their own life satisfaction, depression, and anxiety in 2015, which means the autistic child’s age varies but occurs during childhood for most participants. For each scale, if at least half the items were non-missing, a mean score was calculated and multiplied by the number of items in the scale. All outcome scales showed good to great internal consistency, with ordinal Cronbach’s alpha between α = .76 and α = .94. See Figure 1; see details in Supplementary Table S2 for scales, example items, and Cronbach’s alpha for each outcome measure.

#### Hypothesised Confounders of the Association Between Age at First Diagnosis and Family Well-Being

Based on existing literature, we identified several factors with the potential to confound the relationship between age at diagnosis and family well-being. These were the child’s sex and birth year, parental age at birth, maternal cohabitation status, financial problems, parental educational attainment, household income, maternal parity, world region of origin, county of residence within Norway, social support at ages 3, 8, and 14, maternal concerns at ages 3 and 5, the child’s age at onset of walking, the child’s language skills, social communication, and restricted and repetitive behaviours at age 3, the child’s communication skills and language and motor development at age 5, the child’s externalising behaviours at ages 3 and 5, and co-occurring cerebral palsy, epilepsy, and intellectual disability. We also included a range of polygenic scores (PGS) for traits linked to neurodevelopment and psychiatric conditions. We extracted sex and birth year from the Medical Birth Registry of Norway (MBRN), which is a national health registry containing information about all births in Norway. We used data on co-occurring diagnoses of cerebral palsy, epilepsy, and intellectual disability from the NPR. Finally, we used data from Statistics Norway (SSB) on parental sociodemographic characteristics, including world region of origin, county of residence in Norway, household income, and parental educational attainment. All other variables are from the MoBa questionnaires.

### Polygenic Scores

The MoBa dataset also contains genetic data from mother, father, and child trios (Paltiel et al., 2014). We use genotyped data that have previously undergone phasing, imputation, and quality control procedures, which are outlined in Corfield et al. (2024). Using these data, we estimated PGS based on summary statistics of European samples from the most recent genome-wide association studies (GWAS) of attention-deficit/hyperactivity disorder (ADHD; Demontis et al., 2023), anorexia nervosa (Termorshuizen et al., 2025), anxiety (Purves et al., 2020), autism (Grove et al., 2019), bipolar disorder (Mullins et al., 2021), childhood maltreatment (Warrier et al., 2021), educational attainment (Lee et al., 2018), insomnia (Watanabe et al., 2019), cognitive performance (Savage et al., 2018), depression (Major Depressive Disorder Working Group of the Psychiatric Genomics Consortium, 2025), post-traumatic stress disorder (PTSD; Logue et al., 2015), and schizophrenia (Trubetskoy et al., 2022). These traits were selected as they were associated with age at autism diagnosis in Zhang et al. (2025). Recommended QC of summary statistics was performed prior to estimation of the PGS (Privé et al., 2021).

### Statistical Models

#### Descriptive Analyses

In preliminary descriptive analyses, we quantified associations between our hypothesised confounders and (a) age at first diagnosis and (b) family well-being. We first performed a set of simple linear regression models, incorporating each hypothesised confounder as a single predictor for one outcome from either (a) or (b). We subsequently performed multiple linear regression models, incorporating all hypothesised confounders in a single predictive model.

#### Main Analyses

In our main analyses, we used multiple linear regression models to estimate the extent to which age at autism diagnosis is related to individual family members’ well-being. We used baseline models to first examine the unadjusted association of age at first diagnosis with each well-being outcome. Subsequently, we performed multiple regression using “adjusted” models incorporating all hypothesised confounders. Birth year was included as a technical covariate in all models to account for the systematic link between birth year and timing of the autism diagnosis, as older children have a longer follow-up period than their younger counterparts.

#### Interaction Models

We performed similar adjusted multiple regressions with an interaction term between age of diagnosis and sex (coded 0 for males and 1 for females) to investigate whether the association of age at diagnosis with well-being is different for females than for males. In accordance with recommendations (Yzerbyt et al., 2004), these models additionally incorporated interactions between sex and all hypothesised confounders.

#### Wald Tests

We performed Wald tests to assess whether the beta coefficients for age at first autism diagnosis and for the interaction between age at first autism diagnosis and sex were significantly different between the unadjusted and adjusted models.

### Handling of Missing Data

Based on the observed patterns of missingness, we concluded that the data were missing at random and therefore used multiple imputation to account for missingness in all variables (see Supplementary Materials for a more detailed description of missingness analyses and for the amount of missingness for each variable). We used the MICE R package, version 3.13.0 (Buuren & Groothuis-Oudshoorn, 2011), to perform multiple imputation.

### Multiple Testing Corrections

To account for the potential inflation of Type I error rates due to multiple testing, we performed the Benjamini–Hochberg false discovery rate (FDR)-controlling procedure across all three well-being outcomes for each family member in childhood and in adolescence.

### Software

All analyses were run in R version 4.1.2. We used *phenotools* version 0.4.1 (Hannigan et al., 2021) to prepare variables from MoBa and NPR. We used *genotools* version 0.3.0 (https://github.com/psychgen/genotools/) and *bignspr* version 1.12.18 (Privé et al., 2018) for estimating polygenic scores. All analytic code is available on https://github.com/psychgen/aadx-fam-wellbeing.

### Participatory Methods

In this study, we engaged user representatives from the Norwegian Autism Association (Autismeforeningen) to inform research design and interpretation of results. During the conceptualisation of this study, plans were presented to a group of around 20 autistic adolescents and young adults (diverse in gender) and their carers from all over Norway as part of a 2-day meeting specifically designed for autistic youth (Ungdomstreff). The group provided input, asked questions, and were prompted to think about additional factors that could influence both the age at autism diagnosis and family well-being. Based on their feedback, we made some changes to the list of hypothesised confounders included in our study. On a later occasion, preliminary results were presented to a small panel (*N* = 4) of user representatives during an online user involvement meeting with members of Autismeforeningen who provided feedback, asked questions, and were asked to think of additional explanations for the observed results. To preserve the privacy of this panel, we do not disclose any personal characteristics. Based on their feedback, we added additional potential explanations for our results.

## Results

### Age at Autism Diagnosis in the MoBa Cohort

The average age at diagnosis in years was 12.29 (*SD* = 4.42). Males had a mean age at diagnosis of 11.24 (*SD* = 4.29), whereas females had a mean age at diagnosis of 14.15 (*SD* = 4.03). This is very similar to the mean age at diagnosis of autism in the NPR for the entire Norwegian population born in the same years (*M* = 11.89 [*SD* = 4.31] for males; *M* = 14.24 [*SD* = 4.15] for females). Table 1 shows the sample characteristics for the autism-diagnosed children overall and by earlier, mid and later age at diagnosis. Means and standard deviations for the remaining study variables and mother, father, and sibling outcomes can be found in Figure 1; see details in Supplementary Table S3. Similar tables stratified by sex can be found in Figure 1; see details in Supplementary Tables S4 and S5.

**Table 1.** Sample Characteristics (Means and Standard Deviations (), or Proportion) for Central Clinical and Sociodemographic Variables and Child Outcomes, Overall and Split by Earlier, Mid, and Later Age at Diagnosis.

Variable	Earlier (⩽ 8 years)	Mid (9–14 years)	Later (⩾15 years)	All
n	577 (23.2%)	1,029 (41.4%)	877 (35.3%)	2,483
Female	17.7%	30.1%	55.1%	36%
Age at autism diagnosis	5.9 (1.8)	11.87 (1.65)	16.87 (1.83)	12.59 (4.32)
Age at onset of walking (months)	13.55 (2.31)	13.37 (2.2)	13.19 (2.08)	13.34 (2.18)
ASQ language skills age 3	3.77 (1.24)	4.33 (0.89)	4.56 (0.69)	4.29 (0.96)
SCQ social communication age 3	6.98 (5.38)	3.32 (2.61)	2.78 (2.17)	4.01 (3.77)
SCQ restricted repetitive behaviour age 3	4.89 (2.65)	4.35 (2.62)	4.15 (2.37)	4.41 (2.56)
CBCL externalising behaviour age 3	8.46 (4.07)	6.97 (3.65)	6.21 (3.57)	7.06 (3.82)
CBCL externalising behaviour age 5	8.48 (4.38)	6.00 (3.91)	5.03 (3.8)	6.18 (4.17)
CCC2 communication Coherence age 5	5.99 (3.61)	4.25 (2.97)	3.44 (2.57)	4.3 (3.11)
CDI motor development problems age 5	41.6%	14.5%	6.9%	17.5%
ASQ language development problems age 5	58.3%	24.6%	14.4%	28.1%
Intellectual disability diagnosis	23.4%	5.3%	1.7%	8.3%
Epilepsy diagnosis	12.1%	4.9%	2.9%	5.8%
Cerebral palsy diagnosis	1.6%	1.5%	0.5%	1.1%
Maternal age at birth	29.56 (5.08)	29.75 (5.07)	29.92 (4.75)	29.76 (4.96)
Paternal age at birth	32.59 (5.92)	32.62 (5.92)	32.67 (5.72)	32.63 (5.85)
Maternal years of education	14.08 (2.63)	14.37 (2.58)	14.27 (2.64)	14.27 (2.61)
Paternal years of education	13.8 (2.55)	14.30 (2.77)	14.25 (2.76)	14.16 (2.72)
Parents not cohabiting (18 months)	6.2%	8.5%	5.4%	6.9%
Household income (NOK)	527,379.81 (280,656.05)	550,074.91 (261,730.77)	527,003.42 (240,336.58)	537,351.47 (259,700.33)
Maternal parity	0.62 (0.84)	0.74 (0.91)	0.75 (0.91)	0.72 (0.90)
Parent(s) born in Africa	2.1%	1.3%	0.6%	1.2%
Parent(s) born in Asia	5.7%	3.3%	2.6%	3.6%
Child life enjoyment (8 years)	2.45 (1.00)	2.46 (0.90)	2.70 (0.84)	2.55 (0.91)
Child anxiety (8 years)	1.78 (1.93)	1.58 (1.73)	1.27 (1.42)	1.51 (1.68)
Child depression (8 years)	4.46 (3.75)	4.30 (3.84)	2.94 (3.39)	3.84 (3.72)
Child life satisfaction (14 years)	20.52 (7.06)	18.30 (7.63)	13.83 (8.24)	16.96 (8.21)
Child anxiety (14 years)	1.13 (1.45)	1.99 (2.05)	2.22 (1.95)	1.89 (1.94)
Child depression (14 years)	2.23 (2.68)	3.51 (3.33)	3.86 (3.41)	3.36 (3.28)

*Note*. The thresholds of each age at diagnosis category were based on the timepoints at which the outcomes were measured (i.e., at ages 8 and 14). Although many thresholds exist for a diagnosis to be considered “late,” our thresholds for early and late diagnoses are well below and above the international mean threshold for “late” diagnosis of 11.53 years (A. S. Russell et al., 2025). The variables years of education and household income reflect values from the child’s birth year. The variables parental cohabitation status and financial problems reflect values from when the child was 18 months old. ASQ: Ages and Stages Questionnaire; CBCL: Child Behaviour Checklist; CCC: Children’s Communication Checklist; CDI: Child Development Inventory; SCQ: Social Communication Questionnaire.

In simple regression models, an older age at diagnosis was associated with earlier onset of walking; higher language skills at age 3; fewer social communication difficulties and fewer restricted and repetitive behaviours at age 3; fewer externalising behaviours at ages 3 and 5; lower levels of language and communication difficulties; lower maternal concerns about the child’s development at ages 3 and 5; absence of co-occurring diagnoses of intellectual disability, epilepsy, and cerebral palsy; higher genetic liability to ADHD of the child, female sex, not having a parent born in Asia or Africa, higher maternal parity, and higher levels of social support at ages 3 and 14 (Figure 2, light blue markers). In multiple regression models, where age at diagnosis was predicted by all hypothesised confounders simultaneously, an older age at first diagnosis was associated with lower levels of social communication difficulties, lower maternal concerns about the child’s development at ages 3 and 5, absence of intellectual disability diagnosis, female sex, and higher maternal parity (Figure 2, dark blue markers). Associations between outcome variables and hypothesised confounders can be found in Figure 1; see details in Supplementary Figures S2 to S5.

**Figure 2.**
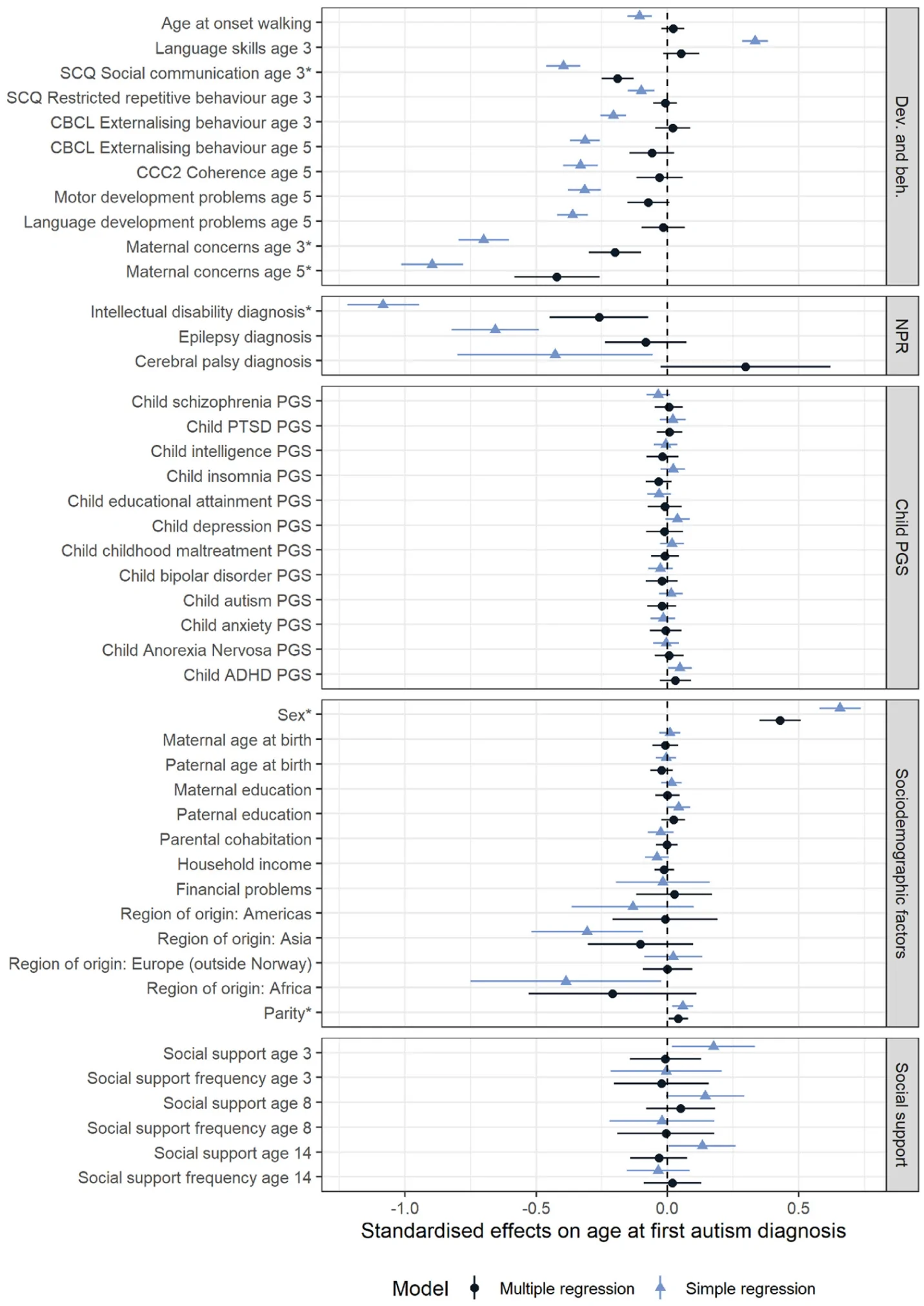
Standardised regression coefficients for all potential confounders on age at first autism diagnosis.

### Associations Between Age at First Diagnosis and Well-Being During Childhood

In baseline models without adjusting for hypothesised confounders, we found that, among the autism-diagnosed children, those who were diagnosed later had higher maternal ratings of well-being in childhood compared to children diagnosed earlier. The later-diagnosed children had higher levels of life satisfaction (β = 0.13, CI: [0.063, 0.190], *p* < .001) and lower levels of anxiety (β = −0.13, CI: [−0.189, −0.074], *p* < .001) and depression (β = −0.18, CI: [−0.241, −0.125], *p* < .001) (Figure 3, light blue lines). We found the same pattern for maternal self-reported well-being measures at the same time point (life satisfaction: β = 0.18, CI: [0.120, 0.231], *p* < .001; anxiety: β = −0.12, CI: [−0.174, −0.060], *p* < .001; depression: β = −0.17, CI: [−0.221, −0.109], *p* < .001; Figure 4, light blue lines). No statistically significant associations were found between age at diagnosis and outcomes in childhood for fathers or siblings (Figure 1; see details in Supplementary Figures S6 and S7, respectively).

**Figure 3.**
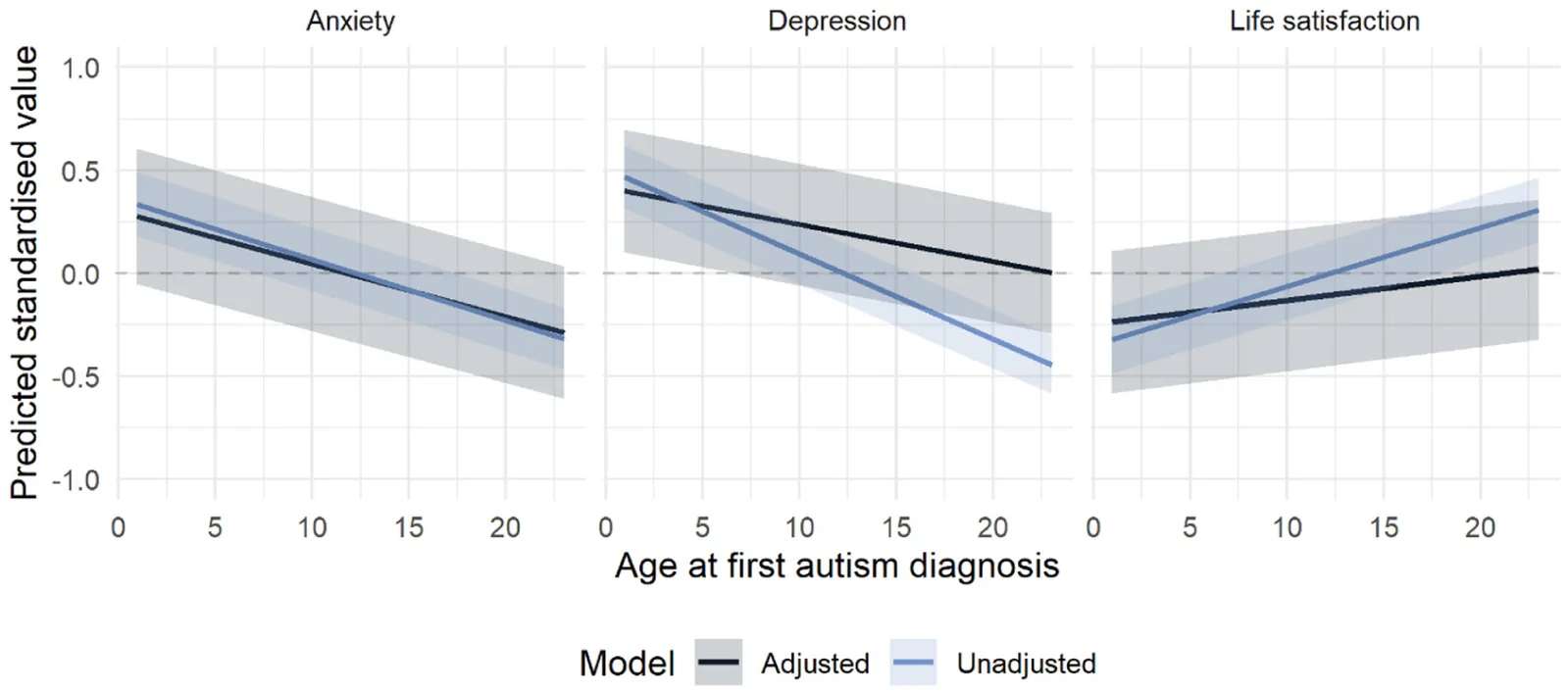
Associations between age at first autism diagnosis and measures of child well-being when the autistic children were 8 years old. *Note*. The bold light blue lines represent the regression slopes for the unadjusted models, whereas the bold dark blue lines represent the regression slopes for the adjusted models. The light and dark ribbons represent the respective 95% confidence intervals.

**Figure 4.**
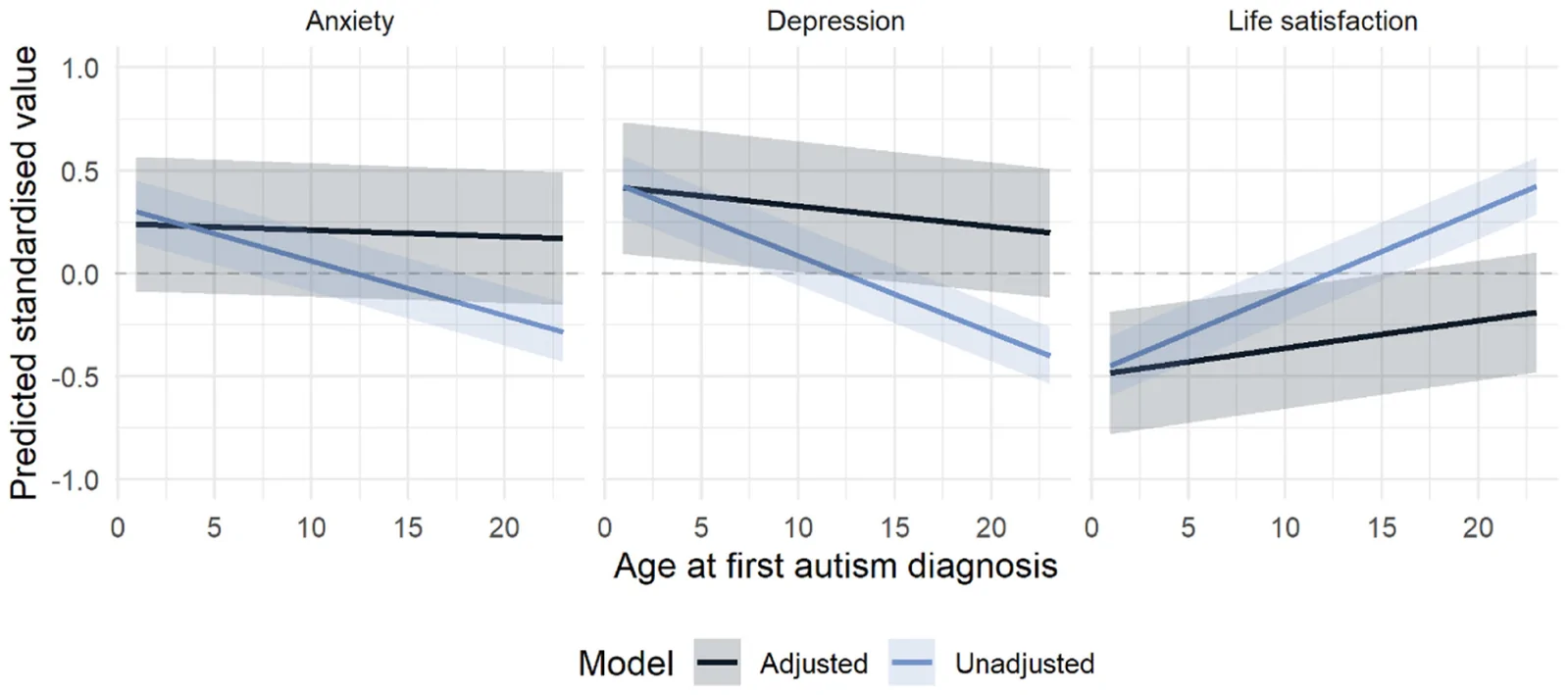
Associations between age at first autism diagnosis and measures of maternal well-being when the autistic children were 8 years old. *Note*. The bold light blue lines represent the regression slopes for the unadjusted models, whereas the bold dark blue lines represent the regression slopes for the adjusted models. The light and dark ribbons represent the respective 95% confidence intervals.

After adjusting for hypothesised confounders, only the association between age at first diagnosis and autistic children’s anxiety remained significant (β = −0.113, CI: [−0.186, −0.040], *p* = .009). All other associations became non-significant relative to the null (Figures 3 and 4, dark blue lines). This change was largely attributable to decreased precision in the adjusted models. Wald tests showed a significant difference between the age at first diagnosis coefficient in the unadjusted and the adjusted models for mother’s life satisfaction (*W* = 6.04, *p* = .021), anxiety (*W* = 5.31, *p* = .021), and depression (*W* = 6.35, *p* = .021), but not for the other outcomes. Specifically, the point estimates for the associations (i.e., the angle of the slopes in Figure 3) with age at diagnosis changed more for mothers’ anxiety (88% reduction) than for mothers’ depression (73.3% reduction) or life satisfaction (66.3% reduction) after adjustment for hypothesised confounders. This suggests that the association between maternal well-being and the child’s age at first diagnosis is significantly impacted by the hypothesised confounders included in this study.

### Associations Between Age at First Diagnosis and Well-Being During Adolescence

For the adolescent autistic children’s own well-being, a direct inversion of the pattern observed for the corresponding childhood outcomes was observed. Specifically, children diagnosed later had lower self-reported life satisfaction (β = −0.24, CI: [−0.328, −0.149], *p* < .001), higher maternally reported anxiety (β = 0.12, CI: [0.061, 0.187], *p* = .001), and higher maternally reported depression (β = 0.13, CI: [0.052, 0.202], *p* = .002). After adjusting for hypothesised confounders, all associations between age at diagnosis and autistic children’s own well-being in adolescence attenuated towards the null (Figure 1; see details in Supplementary Figures S8–S10).

No significant associations were found between age at diagnosis and well-being outcomes at the adolescent time point for mothers or siblings. Wald tests showed no significant differences between the age at first diagnosis coefficients from the unadjusted and adjusted models.

### Interactions With Sex

We found no significant interaction effects between age at first diagnosis and sex in models predicting family well-being in either childhood or adolescence. This indicates that the patterns of association between age at first diagnosis and family well-being are not significantly different between males and females – and, indeed, visual inspection of the effects split by sex confirms that these patterns are highly consistent (Figures 5 and 6; Figure 1; see details in Supplementary Figures S11–S15).

**Figure 5.**
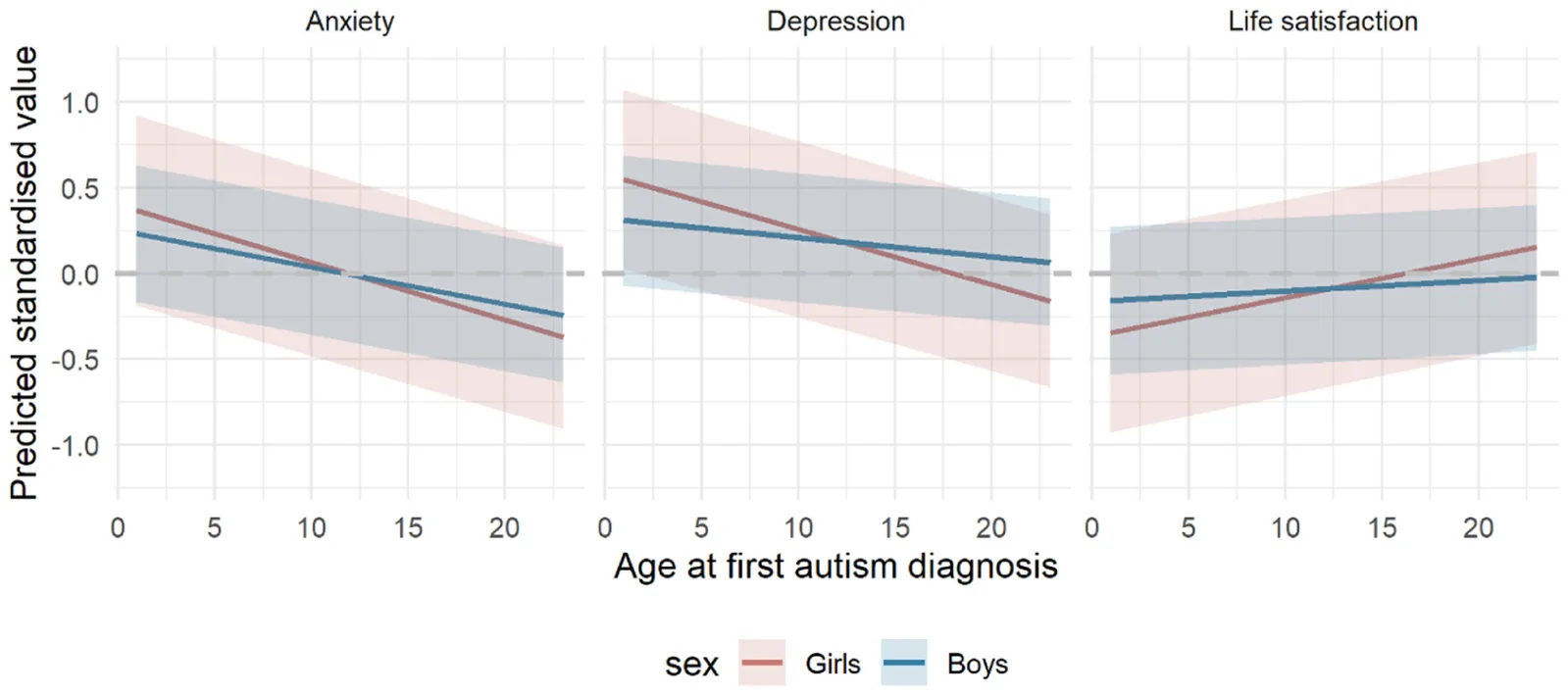
Comparison of the associations between age at first autism and measures of well-being in the autistic children at 8 years old by sex. *Note*. The results are from the adjusted model controlling for all hypothesised confounders. The bold blue lines represent the regression slopes for males, whereas the bold red lines represent the regression slopes for females. The blue and red ribbons represent the respective 95% confidence intervals.

**Figure 6.**
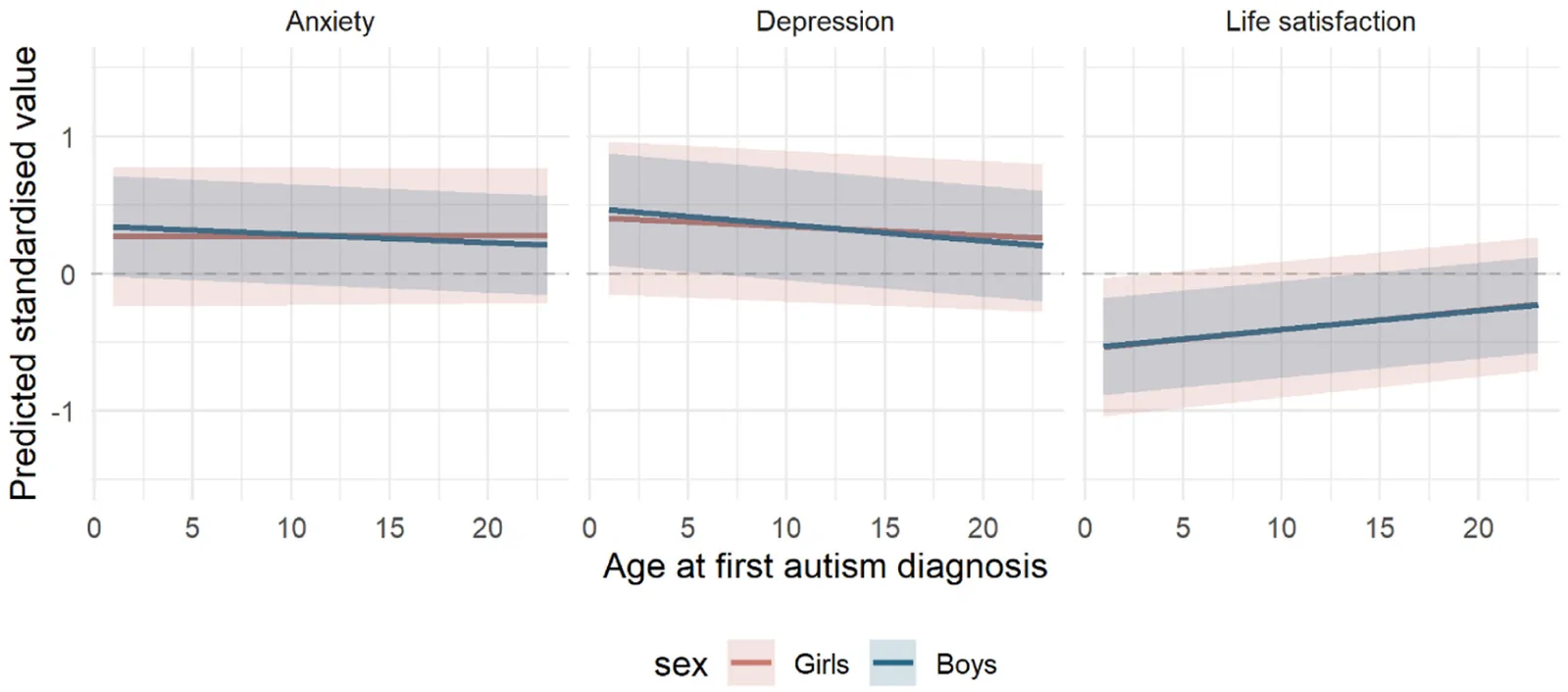
Comparison of the associations between age at first autism and measures of maternal well-being when the autistic children were 8 years old by sex. *Note*. The results are from the adjusted model controlling for all hypothesised confounders. The bold blue lines represent the regression slopes for males, whereas the bold red lines represent the regression slopes for females. The blue and red ribbons represent the respective 95% confidence intervals.

## Discussion

In this study, we characterised the age at autism diagnosis in a prospective population-based cohort sample and investigated the relationship between age at diagnosis and family well-being in both middle childhood and adolescence. We found an average age at diagnosis of 12 years and further identified that age at diagnosis was associated with a range of clinical and demographic factors, including co-occurring diagnoses of intellectual disability, social and communication skills at age 3, and parental region of origin. In preregistered multiple regression analyses, we found significant associations between an earlier autism diagnosis and lower well-being for autistic children and mothers during middle childhood, but these associations decreased and became non-significant after adjusting for our hypothesised confounding variables. This attenuation is consistent with the hypothesis that confounding masks any potential underlying protective effect of earlier diagnosis on family well-being. However, we did observe the inverse relationship in adolescence; that is, earlier autism diagnosis was associated with higher well-being in adolescence. However, the fact that this relationship also attenuated and became non-significant after adjusting for hypothesised confounders suggests this result may be better explained by something other than a protective effect of earlier diagnosis and illustrates the complexities associated with using observational data to investigate the potential impacts of earlier autism diagnoses on well-being.

### Clinical, Demographic, and Genetic Factors Associated With Age at Diagnosis in the MoBa Cohort

Most of the autistic children in our sample were diagnosed in middle childhood or later, with only 23% diagnosed by age 8 years. This is in line with other recent studies showing that autism diagnoses increasingly occur *after* early childhood (Schendel & Thorsteinsson, 2018). This trend has been observed in many European countries, including the United Kingdom (G. Russell et al., 2022) and Sweden (Socialstyrelsen, 2024). We found that age at diagnosis was associated with a range of clinical characteristics of the autistic child – where a later age at diagnosis was associated with lower language, motor, and communication difficulties; fewer restricted and repetitive and externalising behaviours; being a female; lower maternal concerns about development; and absence of co-occurring diagnoses of intellectual disability, epilepsy, and cerebral palsy – and with some family-related factors, where a later age at diagnosis was associated with higher maternal parity, not having a parent born in Asia or Africa, and higher social support in early childhood and adolescence. These findings were also consistent with patterns previously reported in the literature (Daniels & Mandell, 2014; Loubersac et al., 2023). Although age at diagnosis is often also associated with socioeconomic status (Daniels & Mandell, 2014; Loubersac et al., 2023), we did not find any associations between age at diagnosis and household income, financial problems, or parental years of education in the MoBa sample, which could be due to the families with higher education and socioeconomic background being overrepresented in MoBa. Previous literature also suggests an association between age at diagnosis and parental age at birth (Loubersac et al., 2023), but we found no such association in our sample. When mutually adjusting for all factors simultaneously in our models, only lower levels of social communication difficulties, lower maternal concerns about the child’s development in early childhood, the absence of an intellectual disability diagnosis, female sex, and higher maternal parity remained robustly linked to later autism diagnoses, suggesting the particular importance of these factors in the MoBa sample. Notably, parental world region of origin continued to explain a substantial proportion of variation in age at diagnosis even after adjustment for all other factors, but these effects were somewhat imprecisely estimated.

### Associations Between Age at Diagnosis and Family Well-Being

According to the literature, many factors that are associated with receiving an earlier autism diagnosis (Loubersac et al., 2023) are also associated with lower well-being among members of the nuclear family in the context of autism (Tint & Weiss, 2016). Our descriptive analysis confirmed this to be the case for factors such as social communication difficulties, maternal concerns about the child’s development in early childhood, co-occurring intellectual disability diagnosis, sex, and maternal parity. As such, these factors likely contribute to observed relationships between an earlier diagnosis and lower well-being in family members and thus present a challenge for establishing whether or not an earlier diagnosis has a protective effect on family well-being. We set out to investigate the extent of this challenge by assessing the effect of adjusting for a relatively comprehensive set of hypothesised confounding factors independently linked to both age at diagnosis and well-being. The fact that the effects we observed for middle childhood outcomes – where later diagnosis initially predicted *higher* well-being in children and mothers – became non-significant after adjustment indicates that this relationship is, indeed, confounded – and is consistent with the idea that an underlying beneficial effect of earlier diagnosis may be masked in observational data.

An unexpected complexity in our results came from the fact that the pattern we observed for children’s outcomes in *adolescence* was the direct opposite of the pattern of results for outcomes in childhood. In adolescence, an earlier autism diagnosis was observationally associated with *higher* well-being in the unadjusted model. Taken at face value, this relationship would be consistent with a long-term benefit of earlier diagnosis for children’s well-being. However, similar to the results for childhood outcomes, this relationship *decreased* to non-significance after adjusting for all the hypothesised confounders – which runs counter to what would be expected if it represented a true effect of earlier diagnosis. Taken together, these results indicate that both the observational relationship between age at autism diagnosis and well-being and its confounding are more complex than anticipated.

One potential explanation for the pattern of results we observe in adolescence is that, rather than being driven by autistic children experiencing benefits of an early diagnosis by the time they reach adolescence, children experiencing *more* difficulties in adolescence may seek out specialist help where they subsequently receive an autism diagnosis. That is, the association may be underpinned by a link between poorer well-being in adolescence and a subsequent diagnosis, rather than by beneficial impacts of earlier diagnoses in childhood. This explanation is especially plausible, given that a substantial proportion (35.3%) of our sample receives their diagnosis after the age of 14. This explanation is also partially supported by previous literature, as an earlier study examining co-occurring conditions associated with age at first diagnosis showed that a later diagnosis is associated with affective disorders and anxiety (Rødgaard et al., 2021).

Although females were diagnosed later, on average, than males in our sample, we found no interaction effects between sex and age at diagnosis on family well-being in either childhood or adolescence. This suggests that both relationships between age at diagnosis and (family) well-being and the effects of their confounders are similar between (families of) males and females. Other work has shown that sex differences are often explained by other individual-level factors, such as IQ or language level (Kaat et al., 2021).

Taken together, these results show several complexities in both the relationship between age at diagnosis and family well-being and its study using observational data. First, we demonstrate that, as expected, this relationship is confounded by several factors that relate independently to age at diagnosis and aspects of family well-being. Even comprehensive adjustment cannot conclusively determine the nature of this underlying relationship in observational data, since researchers cannot be certain of the extent to which measured covariates capture the “true” confounding that exists in the data. Second, the nature of the relationship between age at diagnosis and family well-being seems to be sensitive to the period in which it is assessed, as highlighted by its inversion in the case of autistic children’s own well-being between childhood and adolescence in our data. This observation is important to consider when seeking to establish whether and when earlier diagnosis exerts a protective effect on family well-being – even when using designs less subject to bias from confounding.

### Study Limitations

When interpreting the results from this study, it is important to consider potential biases that may be due to selection and attrition in MoBa (Biele et al., 2019). For example, in MoBa, there is an overrepresentation of families with higher education levels and socioeconomic backgrounds, and these participants tend to have higher response rates over time (Vejrup et al., 2022). Moreover, it is important to note that many of the variables included in this study came from mother reports. These include the mother-and-child well-being measures that were significantly associated with age at diagnosis, and our reliance on mother reports may also explain why only the relationship between age at diagnosis and well-being in mothers was significantly “corrected” by our adjustment for measured confounders. A further limitation of this study came from independent sources of censoring on our age at diagnosis variable (early diagnoses may have been missing for older children due to NPR data being available only from 2008, and late diagnoses may have been “missing” for younger children who had not yet reached the end of adolescence in the follow-up period). Left-censoring was partially mitigated using supplementary data (from the ABC study, as described in the “Methods” section), and potential bias due to either left- or right-censoring was accounted for by the inclusion of year of birth as a covariate. However, missing diagnoses in the tails may still have reduced the precision of our estimates. Finally, we were limited by the non-inclusion of potentially important confounding factors such as family members’ experience of stigma surrounding autism, as higher stigmatisation could influence both the timing of the diagnosis and family members’ well-being (Okoye et al., 2023). Even for those confounding variables we did include, measurement error would have reduced their effectiveness as statistical controls, which implies that the extent of confounding that we were able to detect is likely an underestimate.

## Conclusion

We examined predictors of age at autism diagnosis and its relationship with family well-being. We found that age at diagnosis is positively associated with child and maternal well-being in childhood but is negatively associated with child well-being in adolescence, although these relationships disappeared after adjusting for a range of clinical, demographic, and genetic factors. We interpret these results to reflect complex patterns of confounding that are difficult to disentangle using observational data. Although observational data are well-suited to finding correlates of both age at diagnosis and family well-being, these data are not suitable for establishing causal links between them. Future studies should aim to make use of other methods and data sources in order to establish the nature of any causal link between the age at diagnosis and family well-being. For example, researchers could leverage interventions aimed at reducing the age at autism diagnosis, such as public awareness campaigns, screening programmes, or changes in clinical practices. Researchers can then compare well-being outcomes for family members of children who received a diagnosis before and after such interventions. These research designs will provide us with a better understanding of the causal mechanisms involved in the relationship between age at autism diagnosis and family well-being.

## Supplemental Material

sj-docx-1-aut-10.1177_13623613261474929 – Supplemental material for Age at Autism Diagnosis in the Norwegian Mother, Father, and Child Cohort Study (MoBa): Exploring the Association With Family Well-Being Supplemental material, sj-docx-1-aut-10.1177_13623613261474929 for Age at Autism Diagnosis in the Norwegian Mother, Father, and Child Cohort Study (MoBa): Exploring the Association With Family Well-Being by Bernt Damian Glaser, Meseret Mamo Bazezew, Daniela Bragantini, Laura Elizabeth Hegemann, Stian Orm, Morgan James Morgan, Somer L. Bishop, Stian Barbo Valand, Laurie John Hannigan and Alexandra Havdahl in Autism

## Data Availability

Data from the Norwegian Mother, Father, and Child Cohort Study is managed by the NIPH. Access requires approval from the Regional Committees for Medical and Health Research Ethics (REC), compliance with the General Data Protection Regulation (GDPR), and data owner approval. Participant consent does not allow individual-level data storage in repositories or journals. Researchers seeking access for replication must apply via www.helsedata.no. All analytic code will be made available on publication via https://github.com/psychgen/aadx-fam-wellbeing.
