# Supplementary material for: Age at Autism Diagnosis in the Norwegian Mother, Father, and Child Cohort Study (MoBa): Exploring the Association With Family Well-Being
Source: Autism. 2026 Aug 22;30(10):2596–609. doi: 10.1177/13623613261474929 (PMC13624353; doi:10.1177/13623613261474929)
Supplement: Supplementary file 1 [file sj-docx-1-aut-10.1177_13623613261474929.docx]

**Exposure variable**

Since NPR data is only available from 2008, follow-up was not available for the older children in MoBa. To account for censoring of the age at first diagnosis we implemented the following pipeline:

1. Since it is unlikely that a child receives an autism diagnosis before the age of 2, the ages of autism diagnosis for children born in 2006 (N = 1101) or later were taken directly from NPR.
2. Of the children born before 2006, many took part in the Autism Birth Cohort (ABC) study (Stoltenberg et al., 2010) in which they received a clinical assessment for autism which resulted in a diagnosis for 134 children. If the clinical assessment occurred prior to the first registered diagnosis in NPR, the age of the child on the date of that clinical assessment would supersede the age at first diagnosis in NPR (n=57).
3. For the remaining children born before 2006, we used their first diagnosis in NPR if it was first registered two years after the registry began in 2008 (see supplementary methods for rationale), which was the case for 1208 children.
4. For the remaining 126 children, age at first diagnosis was set to missing and imputed based on multiple imputation using all analytic variables as described below. The Early Screening of Autistic Traits questionnaire (ESAT; Swinkels et al., 2006) and the Modified Checklist of Autism in Toddlers (M-CHAT; Robins et al., 2001) from the 3 year-old MoBa questionnaire were added as additional predictors for age at first diagnosis. See below for information about the imputation procedure.

**Polygenic score calculation**

If allele frequencies or info scores were provided in the summary statistics, we removed variants with MAF < 0.01 and INFO < 0.6. Additionally, we estimated the SD of the variants from the allele frequencies in the reference panel (sd_ldref) and from on the sample size, beta and SD estimates of the variant from the summary statistics (sd_ss). Variants that had sd_ss < (0.5 * sd_ldref) OR sd_ss > (sd_ldref + 0.1) OR sd_ss < 0.1 OR sd_ldref < 0.05 were removed. PGS were estimated using the “LDpred-auto” model in the LDpred2 software (Privé et al., 2020) We used precomputed LD matrixes based on HapMap3+ variants in the UK Biobank to estimate weights (Privé et al., 2023). The first 20 genomic principal components (PCs), genotype, and imputation batch were regressed on the PGS and the resultant residuals used as the PGS in the models.

**Missing Data**

We examined the extent and patterns of missing data across key study variables and time points. The overall proportion of missing data was approximately 37.88%, with missingness varying across variables from 0% (sex, birth year, maternal parity, and co-occurring diagnoses) to 83.13% (autistic children’s life satisfaction at age 14; see supplementary tables S6 and S7).

To evaluate the nature of missingness, we first ran Little’s (1988) test for Missing Completely at Random (MCAR), which is a chi-square test where the null hypothesis is that the data is MCAR. The MCAR test yielded p < .001, which suggests that the data was not MCAR. We also assessed to what extent missingness in all outcome variables was associated with a subset of key variables (age at autism diagnosis, birth year, social communication difficulties and restricted and repetitive behaviours at age 3, maternal concerns at ages 3 and 5, co-occurring diagnoses, sex, maternal parity, maternal and paternal years of education, county of residence, and household income). Missingness in each outcome variable was significantly predicted by at least one key variable, suggesting that the data is most likely missing at random (MAR).

Missing data were handled using multiple imputation by chained equations (MICE). This approach was chosen because it is appropriate in the presence of missing data under the assumption of MAR (Rubin, 1987), and it has been shown to yield less biased and more efficient estimates than complete-case analysis. We used the mice version 3.13.0 for R to implement the imputation procedure.

| **Deviations** | | | | | |
| --- | --- | --- | --- | --- | --- |
| # | Details | | Original Wording | Deviation Description | Reader Impact |
| 1 | Type | Analysis | *“To test H4.1-4 and H5.1-3, we will perform two sets of multiple regressions with each wellbeing variable for each family member as the dependent variable. In the first set of multiple regressions, each wellbeing variable is predicted by only age at diagnosis. In the second set of regressions, each model will be adjusted for factors a-n.”* | *Due to high right-censoring of the age at first autism diagnosis variable, we included birth year as an instrumental variable, meaning that, in the first set of multiple regressions, outcomes were predicted by age at diagnosis and birth year.* | *The inclusion of birth year as an instrumental variable is aimed at reducing the bias associated with right-censoring in the exposure variable (i.e., older children have a higher age at diagnosis than younger children).* |
|  | Reason | New knowledge |  |  |  |
|  | Timing | After data access |  |  |  |
| 2 | Type | Variables | *“we will perform a multiple regression of age at autism diagnosis as the dependent variable and with the following factors as the independent variable: … m) polygenic scores for … cross-disorder … for all members of the trio.”* | *Due to problems with the summary statistics of the cross-disorder Genome-Wide Association Study (GWAS), we were unable to calculate polygenic scores for all individuals in our study sample.* | *We intended on including the cross-disorder PGS based on a study conducted by Warrier et al. (REF) on the genetic basis for age at autism diagnosis. However, given the number of other hypothesised confounders (including other polygenic scores) included in this study, we suspect that the non-inclusion of the cross-disorder PGS should be of little consequence to the reader.* |
|  | Reason | Plan not possible |  |  |  |
|  | Timing | After data access |  |  |  |

**Table S1.** Preregistration deviation table.

| Variable | Scale | Example item | Response | Cronbach’s α |
| --- | --- | --- | --- | --- |
| Child life satisfaction 8yr | Single item from NHiPIC-30 (Mervielde & De Fruyt, 1999, 2002) | “How well do these statements apply to your child’s behaviour over the past year? – Enjoys life” | 5-point Likert scale: ranging from 1 (“Not very typical”) to 5 (“Very typical”) | NA |
| Child anxiety 8yr | 5-item SCARED (Birmaher et al., 1999) | “My child gets really frightened for no reason at all.” | 3 options: 1 (“Not true”), 2 (“Sometimes true”), 3 (“True”) | .76 |
| Child depression 8yr | 13-item SMFQ (Angold et al., 1995) | “Your child has, in the past two weeks, felt miserable or unhappy” | 3 options: 1 (“Not true”), 2 (“Sometimes true”), 3 (“True”) | .92 |
| Mother life satisfaction 8yr | 5-item SWLS (Diener et al., 1985) | “In most ways, my life is close to my ideal” | 7-point Likert scale: ranging from 1 (“Disagree completely”) to 7 (“Agree completely”) | .94 |
| Mother anxiety 8yr | 4-item SCL anxiety subscale (Derogatis et al., 1973; Tambs & Røysamb, 2014) | “Have you, over the past two weeks been bothered by feeling fearful?” | 4-point Likert scale: ranging from 1 (“Not bothered”) to 4 (“Very bothered”) | .91 |
| Mother depression 8yr | 4-item SCL depression subscale (Derogatis et al., 1973; Tambs & Røysamb, 2014) | “Have you, during the past two weeks, been bothered by feeling blue?” | 4-point Likert scale: ranging from 1 (“Not bothered”) to 4 (“Very bothered”) | .91 |
| Father life satisfaction | 5-item SWLS (Diener et al., 1985) | “In most ways, my life is close to my ideal” | 7-point Likert scale: ranging from 1 (“Disagree completely”) to 7 (“Agree completely”) | .92 |
| Father anxiety | 4-item SCL anxiety subscale (Derogatis et al., 1973; Tambs & Røysamb, 2014) | “Have you, over the past two weeks been bothered by feeling fearful?” | 4-point Likert scale: ranging from 1 (“Not bothered”) to 4 (“Very bothered”) | .90 |
| Father depression 8yr | 4-item SCL depression subscale (Derogatis et al., 1973; Tambs & Røysamb, 2014) | “Have you, during the past two weeks, been bothered by feeling blue?” | 4-point Likert scale: ranging from 1 (“Not bothered”) to 4 (“Very bothered”) | .90 |
| Sibling life satisfaction 8yr | Single item from NHiPIC-30 (Mervielde & De Fruyt, 1999, 2002) | “How well do these statements apply to your child’s behaviour over the past year? – Enjoys life” | 5-point Likert scale: ranging from 1 (“Not very typical”) to 5 (“Very typical”) | NA |
| Sibling anxiety 8yr | 5-item SCARED (Birmaher et al., 1999) | “My child gets really frightened for no reason at all.” | 3 options: 1 (“Not true”), 2 (“Sometimes true”), 3 (“True”) | .78 |
| Sibling depression 8yr | 13-item SMFQ (Angold et al., 1995) | “Your child has, in the past two weeks, felt miserable or unhappy” | 3 options: 1 (“Not true”), 2 (“Sometimes true”), 3 (“True”) | .92 |
| Child life satisfaction 14yr | 5-item SWLS (Diener et al., 1985) | “In most ways, my life is close to my ideal” | 7-point Likert scale: ranging from 1 (“Disagree completely”) to 7 (“Agree completely”) | .94 |
| Child anxiety 14yr | 5-item SCARED (Birmaher et al., 1999) | “My child gets really frightened for no reason at all.” | 3 options: 1 (“Not true”), 2 (“Sometimes true”), 3 (“True”) | .82 |
| Child depression 14yr | 13-item SMFQ (Angold et al., 1995) | “Your child has, in the past two weeks, felt miserable or unhappy” | 3 options: 1 (“Not true”), 2 (“Sometimes true”), 3 (“True”) | .93 |
| Mother life satisfaction 14yr | 5-item SWLS (Diener et al., 1985) | “In most ways, my life is close to my ideal” | 7-point Likert scale: ranging from 1 (“Disagree completely”) to 7 (“Agree completely”) | .94 |
| Mother anxiety 14yr | 5-item SCL anxiety subscale (Derogatis et al., 1973; Tambs & Røysamb, 2014) | “Have you, over the past two weeks been bothered by feeling fearful?” | 4-point Likert scale: ranging from 1 (“Not bothered”) to 4 (“Very bothered”) | .93 |
| Mother depression 14yr | 9-item SCL depression subscale (Derogatis et al., 1973; Tambs & Røysamb, 2014) | “Have you, during the past two weeks, been bothered by feeling blue?” | 4-point Likert scale: ranging from 1 (“Not bothered”) to 4 (“Very bothered”) | .94 |
| Sibling life satisfaction 14yr | 5-item SWLS (Diener et al., 1985) | “In most ways, my life is close to my ideal” | 7-point Likert scale: ranging from 1 (“Disagree completely”) to 7 (“Agree completely”) | .94 |
| Sibling anxiety 14yr | 5-item SCARED (Birmaher et al., 1999) | “My child gets really frightened for no reason at all.” | 3 options: 1 (“Not true”), 2 (“Sometimes true”), 3 (“True”) | .87 |
| Sibling depression 14yr | 13-item SMFQ (Angold et al., 1995) | “Your child has, in the past two weeks, felt miserable or unhappy” | 3 options: 1 (“Not true”), 2 (“Sometimes true”), 3 (“True”) | .94 |

**Table S2.** Scales, example items, response options, and Cronbach’s alpha for all outcome variables. NHiPIC-30 = Short Norwegian Hierarchical Personality Inventory for Children; SCARED = Screen for Child Anxiety Related Disorders; SCL = Hopkins Symptoms Checklist; SMFQ = Short Mood and Feelings Questionnaire; SWLS = Satisfaction with Life Scale.

| Variable | Earlier  (≤ 8 years) | Mid  (9-14 years) | Later  (≥15 years) | All |
| --- | --- | --- | --- | --- |
| Financial problems | 0.42 (0.75) | 0.46 (0.78) | 0.45 (0.77) | 0.45 (0.77) |
| Region of origin: Americas | 3.1% | 3.4% | 2.4% | 3.0 % |
| Region of origin: Europe (outside Norway) | 14.4% | 14.5% | 17 % | 15.3% |
| Region of origin: Norway | 74.7% | 77.6% | 77.1% | 76.7% |
| Social support age 3 | 1.44 (0.63) | 1.48 (0.57) | 1.5 (0.58) | 1.48 (0.59) |
| Social support frequency age 3 | 1.68 (0.5) | 1.67 (0.51) | 1.69 (0.51) | 1.68 (0.51) |
| Social support age 8 | 1.41 (0.64) | 1.34 (0.59) | 1.4 (0.57) | 1.38 (0.6) |
| Social support frequency age 8 | 0.42 (0.57) | 0.41 (0.57) | 0.46 (0.58) | 0.43 (0.57) |
| Social support age 14 | 1.27 (0.64) | 1.27 (0.61) | 1.29 (0.65) | 1.27 (0.63) |
| Social support frequency age 14 | 0.59 (0.63) | 0.69 (0.67) | 0.67 (0.66) | 0.66 (0.66) |
| Child ADHD PGS | 0.06 (0.97) | 0.16 (1.04) | 0.21 (0.98) | 0.16 (1.01) |
| Child Anorexia Nervosa PGS | 0.12 (0.97) | 0.13 (0.98) | 0.13 (0.98) | 0.13 (0.98) |
| Child anxiety PGS | 0.17 (0.98) | 0.19 (1.02) | 0.15 (0.98) | 0.17 (1) |
| Child autism PGS | 0.36 (0.94) | 0.33 (1.06) | 0.35 (1.02) | 0.34 (1.02) |
| Child bipolar disorder PGS | 0.17 (0.98) | 0.14 (1.02) | 0.11 (1) | 0.14 (1) |
| Child childhood maltreatment PGS | 0.21 (0.94) | 0.26 (1.02) | 0.25 (0.99) | 0.24 (0.99) |
| Child educational attainment PGS | 0.24 (0.98) | 0.13 (1.02) | 0.1 (0.99) | 0.14 (1) |
| Child insomnia PGS | -0.06 (0.98) | 0.08 (1) | 0.06 (0.99) | 0.04 (0.99) |
| Child intelligence PGS | 0.18 (1.01) | 0.16 (1.03) | 0.14 (0.99) | 0.16 (1.01) |
| Child depression PGS | 0.17 (1) | 0.27 (1) | 0.28 (0.97) | 0.25 (0.99) |
| Child PTSD PGS | 0.03 (0.98) | 0 (0.98) | 0.06 (0.97) | 0.03 (0.98) |
| Child schizophrenia PGS | 0.19 (0.97) | 0.1 (1.01) | 0.14 (1.03) | 0.13 (1.01) |
| Mother ADHD PGS | 0.06 (1) | 0.17 (1.02) | 0.17 (1) | 0.14 (1.01) |
| Mother Anorexia Nervosa PGS | 0.1 (1) | 0.11 (1.03) | 0.09 (0.98) | 0.1 (1) |
| Mother anxiety PGS | 0.16 (0.96) | 0.09 (1) | 0.13 (1.01) | 0.12 (1) |
| Mother autism PGS | 0.13 (1.03) | 0.22 (1.01) | 0.25 (1.04) | 0.21 (1.03) |
| Mother bipolar disorder PGS | 0.15 (1.03) | 0.1 (1.03) | 0.02 (1.03) | 0.08 (1.03) |
| Mother childhood maltreatment PGS | 0.09 (1.01) | 0.09 (1) | 0.21 (1) | 0.13 (1) |
| Mother educational attainment PGS | 0.06 (1.02) | 0.04 (1) | 0.05 (0.98) | 0.05 (1) |
| Mother insomnia PGS | -0.08 (0.98) | 0.03 (0.97) | 0.07 (0.99) | 0.02 (0.98) |
| Mother intelligence PGS | 0.03 (1.02) | 0.12 (1.05) | 0.09 (1.03) | 0.09 (1.04) |
| Mother depression PGS | 0.12 (1.01) | 0.18 (0.98) | 0.22 (0.98) | 0.18 (0.98) |
| Mother PTSD PGS | 0.04 (0.99) | -0.02 (0.98) | 0 (0.94) | 0 (0.97) |
| Mother schizophrenia PGS | 0.22 (0.95) | 0.11 (1.02) | 0.02 (1.05) | 0.1 (1.02) |
| Father ADHD PGS | 0.05 (1) | 0.08 (1.01) | 0.09 (1.04) | 0.08 (1.02) |
| Father Anorexia Nervosa PGS | 0.03 (0.95) | 0.06 (1.02) | 0.05 (0.95) | 0.05 (0.98) |
| Father anxiety PGS | 0.12 (1.01) | 0.08 (0.99) | 0.01 (0.94) | 0.07 (0.98) |
| Father autism PGS | 0.22 (1.02) | 0.15 (1.06) | 0.09 (0.98) | 0.14 (1.02) |
| Father bipolar disorder PGS | 0.02 (1) | -0.03 (0.97) | 0.09 (1.02) | 0.02 (0.99) |
| Father childhood maltreatment PGS | 0.07 (0.97) | 0.14 (1.02) | 0.19 (0.95) | 0.14 (0.99) |
| Father educational attainment PGS | 0.09 (1.02) | 0.16 (1.03) | 0.11 (1.03) | 0.13 (1.03) |
| Father insomnia PGS | 0.05 (1.02) | 0.04 (1.01) | 0.09 (0.99) | 0.06 (1) |
| Father intelligence PGS | 0.07 (1.04) | 0.14 (0.99) | 0.14 (0.96) | 0.13 (0.99) |
| Father depression PGS | 0.11 (0.93) | 0.06 (1.05) | 0.13 (0.96) | 0.1 (0.99) |
| Father PTSD PGS | -0.02 (0.89) | -0.01 (1) | 0.06 (0.92) | 0.01 (0.95) |
| Father schizophrenia PGS | 0.03 (0.98) | -0.08 (1.04) | 0.18 (0.96) | 0.03 (1.01) |

**Table S3**. Sample characteristics (means and standard deviations (), or proportion) for the remaining hypothesised confounding variables, overall and split by earlier, mid, and later age at diagnosis. The financial problems variable reflects values from when the child was 18 months old.

| Variable | Earlier  (≤ 8 years) | Mid  (9-14 years) | Later  (≥15 years) | All |
| --- | --- | --- | --- | --- |
| Mother life satisfaction 8yr | 19.49 (6.68) | 20.8 (6.19) | 21.69 (6.06) | 20.83 (6.3) |
| Mother anxiety 8yr | 1.52 (2.01) | 1.29 (1.81) | 1.26 (1.92) | 1.33 (1.9) |
| Mother depression 8 yr | 2.59 (2.73) | 2.34 (2.51) | 1.84 (2.22) | 2.22 (2.48) |
| Father life satisfaction | 21.46 (5.96) | 20.79 (5.93) | 21.32 (6.06) | 21.12 (5.98) |
| Father anxiety | 0.84 (1.43) | 1.05 (1.71) | 0.94 (1.63) | 0.96 (1.62) |
| Father depression 8yr | 1.34 (1.79) | 1.72 (2.09) | 1.46 (2.19) | 1.54 (2.06) |
| Sibling life satisfaction 8yr | 2.74 (0.7) | 2.95 (0.78) | 3.05 (0.71) | 2.95 (0.75) |
| Sibling anxiety 8yr | 1.29 (1.49) | 1.09 (1.44) | 1.13 (1.09) | 1.15 (1.33) |
| Sibling depression 8yr | 2.28 (2.63) | 2.12 (2.61) | 2.41 (3.14) | 2.26 (2.82) |
| Mother life satisfaction 14yr | 18.74 (7.18) | 18.03 (7.34) | 18.88 (7.3) | 18.49 (7.29) |
| Mother anxiety 14yr | 1.97 (2.7) | 2.06 (2.62) | 2.2 (2.49) | 2.09 (2.59) |
| Mother depression 14yr | 6.14 (6.23) | 6.63 (6.18) | 6.87 (6.29) | 6.61 (6.22) |
| Sibling life satisfaction 14yr | 20.33 (6.35) | 21.16 (6.78) | 18.88 (7.44) | 20.24 (6.98) |
| Sibling anxiety 14yr | 0.97 (1.7) | 0.78 (1.3) | 1 (1.33) | 0.89 (1.39) |
| Sibling depression 14yr | 1.68 (2.58) | 1.52 (2.11) | 2 (2.66) | 1.72 (2.4) |

**Table S3**. Sample characteristics (means and standard deviations (), or proportion) for the remaining outcome variables, overall and split by earlier, mid, and later age at diagnosis. The father outcomes were all measured in 2015. Mothers’ outcomes were measured when the autistic child was 8 years old or 14 years old, siblings’ outcomes were measured when the sibling was 8 or 14 years old.

| Variable | Earlier  (≤ 8 years) | Mid  (9-14 years) | Later  (≥15 years) | All |
| --- | --- | --- | --- | --- |
| n | 475 (29.9%) | 719 (45.3%) | 394 (24.8%) | 1588 |
| Age at autism diagnosis | 5.9 (1.77) | 11.67 (1.66) | 16.68 (1.81) | 11.57 (4.23) |
| Age at onset walking (months) | 13.47 (2.25) | 13.33 (2.16) | 13.23 (2.04) | 13.35 (2.16) |
| ASQ Language skills age 3 | 3.74 (1.22) | 4.27 (0.89) | 4.44 (0.77) | 4.16 (1.01) |
| SCQ Social communication age 3 | 6.9 (5.35) | 3.61 (2.64) | 3.15 (2.65) | 4.53 (4.04) |
| SCQ Restricted repetitive behaviour age 3 | 5.03 (2.68) | 4.49 (2.66) | 4.39 (2.41) | 4.63 (2.62) |
| CBCL Externalising behaviour age 3 | 8.45 (4.12) | 6.99 (3.59) | 6.54 (3.67) | 7.34 (3.86) |
| CBCL Externalising behaviour age 5 | 8.5 (4.42) | 5.99 (3.83) | 5.17 (4) | 6.54 (4.24) |
| CCC2 Communication Coherence age 5 | 5.95 (3.55) | 4.43 (3) | 3.62 (2.36) | 4.68 (3.15) |
| CDI Motor development problems age 5 | 38.6% | 16 % | 8.4% | 20.9% |
| ASQ Language development problems age 5 | 58.2% | 28.5% | 21.3% | 35.5% |
| Intellectual disability diagnosis | 19.8% | 5.7% | 2.8% | 9.2% |
| Epilepsy diagnosis | 10.7% | 4.3% | 4.1% | 6.2% |
| Cerebral palsy diagnosis | - | - | - | 1.1% |
| Maternal age at birth | 29.5 (5.07) | 29.67 (5.1) | 29.98 (4.78) | 29.69 (5.01) |
| Paternal age at birth | 32.65 (6.01) | 32.53 (5.9) | 32.5 (5.82) | 32.56 (5.91) |
| Maternal years of education | 14.12 (2.63) | 14.31 (2.54) | 14.12 (2.63) | 14.2 (2.59) |
| Paternal years of education | 13.86 (2.55) | 14.2 (2.75) | 14.14 (2.83) | 14.09 (2.71) |
| Parents not cohabiting (18m) | 6.4% | 9.6% | 3.5% | 7.1% |
| Household income (NOK) | 515 606.94 (181 805.14) | 543 592.59 (254 613.94) | 523 935.74 (206 375.63) | 530 748.90 (224 371.09) |
| Maternal parity | 0.62 (0.84) | 0.73 (0.92) | 0.73 (0.88) | 0.7 (0.89) |
| Parent(s) born in Africa | - | - | - | 1.3% |
| Parent(s) born in Asia | - | - | - | 4.2% |
| Child life enjoyment 8yr | 2.43 (1) | 2.44 (0.86) | 2.56 (0.83) | 2.46 (0.9) |
| Child anxiety 8yr | 1.67 (1.77) | 1.57 (1.74) | 1.12 (1.14) | 1.5 (1.64) |
| Child depression 8yr | 4.5 (3.87) | 4.36 (3.66) | 3.11 (3.41) | 4.11 (3.7) |
| Child life satisfaction 14yr | 20.7 (7.37) | 19.53 (7.38) | 17.15 (7.75) | 19.38 (7.54) |
| Child anxiety 14yr | 1.99 (2.84) | 2.23 (2.86) | 2.23 (2.37) | 2.16 (2.75) |
| Child depression 14yr | 6.27 (6.31) | 7.12 (6.62) | 6.66 (6.55) | 6.76 (6.5) |

**Table S4**. Sample characteristics (means and standard deviations (), or proportion) for central clinical and sociodemographic variables and child outcomes, overall and split by earlier, mid, and later age at diagnosis for autistic males in the sample. The thresholds of each age at diagnosis category were based on the timepoints at which the outcomes were measured (i.e., at ages 8 and 14). The variables years of education and household income reflect values from the child’s birth year. Values that indicate 10 or fewer individuals or allow for the calculation of such values have been removed. The variables parental cohabitation status and financial problems reflect values from when the child was 18 months old. ASQ=Ages and Stages Questionnaire; CBCL = Child Behaviour Checklist; CCC = Children’s Communication Checklist; CDI = Child Development Inventory; SCQ = Social Communication Questionnaire.

| Variable | Earlier  (≤ 8 years) | Mid  (9-14 years) | Later  (≥15 years) | All |
| --- | --- | --- | --- | --- |
| n | 102 (11.4%) | 310 (34.6%) | 483 (54%) | 895 |
| Age at autism diagnosis | 5.9 (1.92) | 12.33 (1.53) | 17.02 (1.84) | 14.3 (3.91) |
| Age at onset walking (months) | 14.05 (2.63) | 13.45 (2.29) | 13.16 (2.1) | 13.34 (2.23) |
| ASQ Language skills age 3 | 14.05 (2.63) | 13.45 (2.29) | 13.16 (2.1) | 13.34 (2.23) |
| SCQ Social communication age 3 | 7.32 (5.51) | 2.65 (2.4) | 2.53 (1.7) | 3.16 (3.1) |
| SCQ Restricted repetitive behaviour age 3 | 4.31 (2.44) | 4.03 (2.5) | 3.98 (2.33) | 4.03 (2.4) |
| CBCL Externalising behaviour age 3 | 8.49 (3.88) | 6.92 (3.79) | 5.97 (3.48) | 6.58 (3.72) |
| CBCL Externalising behaviour age 5 | 8.37 (4.23) | 6.02 (4.07) | 4.93 (3.69) | 5.63 (3.99) |
| CCC2 Communication Coherence age 5 | 6.25 (4.04) | 3.88 (2.91) | 3.34 (2.69) | 3.73 (2.96) |
| CDI Motor development problems age 5 | 57.7% | 11.3% | 5.9% | 12.2% |
| ASQ Language development problems age 5 | 59.3% | 16.5% | 10.1 % | 16.7% |
| Intellectual disability diagnosis | 40.2% | - | - | 6.6% |
| Epilepsy diagnosis | 18.6% | - | - | 5.3% |
| Cerebral palsy diagnosis | - | - | - | - |
| Maternal age at birth | 29.83 (5.15) | 29.93 (5.02) | 29.87 (4.72) | 29.89 (4.87) |
| Paternal age at birth | 32.32 (5.53) | 32.83 (5.98) | 32.81 (5.64) | 32.76 (5.74) |
| Maternal years of education | 13.89 (2.6) | 14.52 (2.68) | 14.39 (2.65) | 14.38 (2.65) |
| Paternal years of education | 13.49 (2.54) | 14.52 (2.79) | 14.33 (2.71) | 14.3 (2.73) |
| Parents not cohabiting (18m) | - | - | - | 6.4% |
| Household income (NOK) | 585 571.43 (551 681.58) | 563 486.59 (275 892.45) | 529 283.58 (263 034.05) | 548 603.60 (310 656.84) |
| Maternal parity | 0.64 (0.85) | 0.76 (0.89) | 0.76 (0.93) | 0.75 (0.91) |
| Parent(s) born in Africa | - | - | - | - |
| Parent(s) born in Asia | - | - | - | 2.6% |
| Child life enjoyment 8yr | 2.43 (1) | 2.44 (0.86) | 2.56 (0.83) | 2.46 (0.9) |
| Child anxiety 8yr | 1.67 (1.77) | 1.57 (1.74) | 1.12 (1.14) | 1.5 (1.64) |
| Child depression 8yr | 4.5 (3.87) | 4.36 (3.66) | 3.11 (3.41) | 4.11 (3.7) |
| Child life satisfaction 14yr | 20.7 (7.37) | 19.53 (7.38) | 17.15 (7.75) | 19.38 (7.54) |
| Child anxiety 14yr | 1.12 (1.47) | 1.75 (1.93) | 1.66 (1.56) | 1.54 (1.74) |
| Child depression 14yr | 2.2 (2.7) | 3.1 (3.33) | 2.87 (2.86) | 2.78 (3.07) |

**Table S5**. Sample characteristics (means and standard deviations (), or proportion) for central clinical and sociodemographic variables and child outcomes, overall and split by earlier, mid, and later age at diagnosis for autistic females in the sample. The thresholds of each age at diagnosis category were based on the timepoints at which the outcomes were measured (i.e., at ages 8 and 14). Values that indicate 10 or fewer individuals or allow for the calculation of such values have been removed. The variables years of education and household income reflect values from the child’s birth year. The variables parental cohabitation status and financial problems reflect values from when the child was 18 months old. ASQ=Ages and Stages Questionnaire; CBCL = Child Behaviour Checklist; CCC = Children’s Communication Checklist; CDI = Child Development Inventory; SCQ = Social Communication Questionnaire.

| Variable | Percentage missingness |
| --- | --- |
| Child life satisfaction 8yr | 66.25% |
| Child anxiety 8yr | 65.93% |
| Child depression 8yr | 65.93% |
| Mother life satisfaction 8yr | 66.94% |
| Mother anxiety 8yr | 66.77% |
| Mother depression 8 yr | 66.73% |
| Father life satisfaction | 73.26% |
| Father anxiety | 73.1% |
| Father depression 8yr | 73.14% |
| Sibling life satisfaction 8yr | 63.2% |
| Sibling anxiety 8yr | 62.96% |
| Sibling depression 8yr | 62.84% |
| Child life satisfaction 14yr | 83.13% |
| Child anxiety 14yr | 78.65% |
| Child depression 14yr | 79.26% |
| Mother life satisfaction 14yr | 78.21% |
| Mother anxiety 14yr | 78.17% |
| Mother depression 14yr | 78.17% |
| Sibling life satisfaction 14yr | 79.22% |
| Sibling anxiety 14yr | 75.18% |
| Sibling depression 14yr | 75.79% |

**Table S6.** Percentage missingness for each outcome variable.

| Variable | Percentage missingness |
| --- | --- |
| Age at autism diagnosis | 5.07% |
| Sex | 0% |
| Birth year | 0% |
| Parity | 0% |
| Maternal age at birth | 0.12% |
| Paternal age at birth | 0.85% |
| Parental cohabitation | 34.76% |
| Financial problems | 37.94% |
| Maternal concerns age 3 | 51.39% |
| Maternal concerns age 5 | 68.87% |
| Social support age 3 | 50.79% |
| Social support frequency age 3 | 51.19% |
| Social support age 8 | 66.77% |
| Social support frequency age 8 | 66.85% |
| Social support age 14 | 78.13% |
| Social support frequency age 14 | 78.21% |
| Age at onset walking | 39.35% |
| Language skills age 3 | 49.5% |
| SCQ Social communication age 3 | 48.09% |
| SCQ Restricted repetitive behaviour age 3 | 48.17% |
| CBCL Externalising behaviour age 3 | 48.37% |
| Motor development problems age 5 | 67.98% |
| Language development problems age 5 | 67.94% |
| CCC2 Coherence age 5 | 69.03% |
| CBCL Externalising behaviour age 5 | 67.66% |
| Cerebral palsy diagnosis | 0% |
| Epilepsy diagnosis | 0% |
| Intellectual disability diagnosis | 0% |
| Household income | 27.47% |
| County | 0.12% |
| Parental world region of origin | 0.12% |
| Maternal education | 1.41% |
| Paternal education | 25.33% |
| Child ADHD PGS | 35.16% |
| Child Anorexia Nervosa PGS | 35.16% |
| Child anxiety PGS | 35.16% |
| Child autism PGS | 35.16% |
| Child bipolar disorder PGS | 35.16% |
| Child childhood maltreatment PGS | 35.16% |
| Child educational attainment PGS | 35.16% |
| Child insomnia PGS | 35.16% |
| Child intelligence PGS | 35.16% |
| Child depression PGS | 35.16% |
| Child PTSD PGS | 35.16% |
| Child schizophrenia PGS | 35.16% |
| Mother ADHD PGS | 17.36% |
| Mother Anorexia Nervosa PGS | 17.36% |
| Mother anxiety PGS | 17.36% |
| Mother autism PGS | 17.36% |
| Mother bipolar disorder PGS | 17.36% |
| Mother childhood maltreatment PGS | 17.36% |
| Mother educational attainment PGS | 17.36% |
| Mother insomnia PGS | 17.36% |
| Mother intelligence PGS | 17.36% |
| Mother depression PGS | 17.36% |
| Mother PTSD PGS | 17.36% |
| Mother schizophrenia PGS | 17.36% |
| Father ADHD PGS | 44.06% |
| Father Anorexia Nervosa PGS | 44.06% |
| Father anxiety PGS | 44.06% |
| Father autism PGS | 44.06% |
| Father bipolar disorder PGS | 44.06% |
| Father childhood maltreatment PGS | 44.06% |
| Father educational attainment PGS | 44.06% |
| Father insomnia PGS | 44.06% |
| Father intelligence PGS | 44.06% |
| Father depression PGS | 44.06% |
| Father PTSD PGS | 44.06% |
| Father schizophrenia PGS | 44.06% |
| Sibling ADHD PGS | 31.05% |
| Sibling Anorexia Nervosa PGS | 31.05% |
| Sibling anxiety PGS | 31.05% |
| Sibling autism PGS | 31.05% |
| Sibling bipolar disorder PGS | 31.05% |
| Sibling childhood maltreatment PGS | 31.05% |
| Sibling educational attainment PGS | 31.05% |
| Sibling insomnia PGS | 31.05% |
| Sibling intelligence PGS | 31.05% |
| Sibling depression PGS | 31.05% |
| Sibling PTSD PGS | 31.05% |
| Sibling schizophrenia PGS | 31.05% |
| Age difference with sibling | 0.24% |

**Table S7.** Percentage missingness for age at autism diagnosis and all covariates.

|  | Child | | Father | | | Mother | | Sibling | |
| --- | --- | --- | --- | --- | --- | --- | --- | --- | --- |
| Outcome | **Unadjusted** | **Adjusted** | **Unadjusted** | **Adjusted** | | **Unadjusted** | **Adjusted** | **Unadjusted** | **Adjusted** |
| Childhood Anxiety | -0.13 [-0.19, -0.07] *** | -0.11 [-0.19, -0.04]** | -0.04 [-0.09, 0.02] | -0.00 [-0.08, 0.07] | -0.12 [-0.17, -0.06] *** | | -0.01 [-0.08, 0.05] | -0.07 [-0.16, 0.02] | -0.04 [-0.15, 0.07] |
| Childhood Depression | -0.18 [-0.24, -0.12 ] *** | -0.08 [-0.15, -0.01]† | -0.04 [-0.09, 0.02] | 0.00 [-0.06, 0.07] | -0.16 [-0.22, -0.11] *** | | -0.04 [-0.12, 0.03] | -0.00 [-0.10, 0.09] | 0.01 [-0.10, 0.12] |
| Childhood Life satisfaction | 0.13 [0.06, 0.19] *** | 0.05 [-0.02, 0.12] | 0.04 [-0.01, 0.10] | -0.00 [-0.06, 0.06] | 0.17 [0.12, 0.23] *** | | 0.06 [-0.01, 0.13] | 0.13 [0.02, 0.24]† | 0.05 [-0.07, 0.18] |
| Adolescent Anxiety | 0.12 [0.06, 0.19] *** | 0.06 [-0.02, 0.14] | NA | NA | -0.06 [-0.12, 0.00]† | | 0.02 [-0.06, 0.10] | 0.05 [-0.05, 0.14] | -0.02 [-0.14, 0.10] |
| Adolescent Depression | 0.13 [0.05, 0.20] ** | 0.06 [-0.04, 0.16] | NA | NA | -0.06 [-0.12, -0.01]† | | 0.03 [-0.04, 0.09] | 0.05 [-0.04, 0.14] | -0.01 [-0.12, 0.10] |
| Adolescent Life satisfaction | -0.24 [-0.33, -0.15]*** | -0.12 [-0.24, 0.00] | NA | NA | 0.06 [0.00, 0.11]† | | -0.00 [-0.07, 0.06] | -0.08 [-0.18, 0.03] | -0.07 [-0.17, 0.04] |

**Table S8**. Beta coefficients [and 95% CIs] for age at autism diagnosis on wellbeing outcomes for each family member for our main analyses. Asterisks and crosses indicate p-values after Benjamini-Hochberg FDR-adjustment: *** p < .001, ** p < .01, * p < .05, † p < .1.

|  |  | Child | | Father | | Mother | | Sibling | |
| --- | --- | --- | --- | --- | --- | --- | --- | --- | --- |
| Outcome | **Term** | **Unadjusted** | **Adjusted** | **Unadjusted** | **Adjusted** | **Unadjusted** | **Adjusted** | **Unadjusted** | **Adjusted** |
| Childhood Anxiety | **Mid vs Early** | -0.19 [-0.33, -0.05]* | -0.14 [-0.28, 0.01] | -0.18 [-0.34, -0.02]* | -0.02 [-0.19, 0.15] | -0.03 [-0.17, 0.12] | 0.03 [-0.13, 0.20] | -0.11 [-0.36, 0.14] | -0.03 [-0.28, 0.22] |
| Childhood Anxiety | **Late vs Early** | -0.32 [-0.47, -0.17]*** | -0.24 [-0.42, -0.07]* | -0.28 [-0.44, -0.13]** | -0.02 [-0.21, 0.16] | -0.07 [-0.23, 0.08] | 0.02 [-0.16, 0.21] | -0.15 [-0.41, 0.10] | -0.04 [-0.34, 0.25] |
| Childhood Depression | **Mid vs Early** | -0.20 [-0.34, -0.05]* | -0.05 [-0.19, 0.09] | -0.22 [-0.35, -0.08]** | -0.04 [-0.18, 0.11] | -0.02 [-0.16, 0.12] | 0.06 [-0.09, 0.21] | -0.00 [-0.25, 0.24] | -0.04 [-0.29, 0.22] |
| Childhood Depression | **Late vs Early** | -0.44 [-0.60, -0.29]*** | -0.16 [-0.36, 0.03] | -0.41 [-0.55, -0.26]*** | -0.10 [-0.28, 0.08] | -0.08 [-0.22, 0.07] | 0.04 [-0.14, 0.22] | -0.02 [-0.27, 0.23] | -0.00 [-0.29, 0.28] |
| Childhood Life satisfaction | **Mid vs Early** | 0.11 [-0.02, 0.25] | 0.03 [-0.12, 0.18] | 0.26 [0.12, 0.39]** | 0.07 [-0.06, 0.21] | 0.04 [-0.09, 0.17] | -0.04 [-0.17, 0.10] | 0.21 [-0.04, 0.47] | 0.10 [-0.14, 0.33] |
| Childhood Life satisfaction | **Late vs Early** | 0.30 [0.13, 0.46]** | 0.10 [-0.09, 0.28] | 0.43 [0.29, 0.58]*** | 0.13 [-0.05, 0.30] | 0.11 [-0.03, 0.24] | -0.02 [-0.17, 0.13] | 0.38 [0.08, 0.67]* | 0.16 [-0.15, 0.47] |
| Adolescent Anxiety | **Mid vs Early** | 0.17 [0.03, 0.30]* | 0.10 [-0.04, 0.24] | -0.09 [-0.23, 0.05] | 0.02 [-0.12, 0.17] | NA | NA | 0.02 [-0.22, 0.26] | -0.06 [-0.31, 0.18] |
| Adolescent Anxiety | **Late vs Early** | 0.30 [0.14, 0.47]** | 0.14 [-0.06, 0.34] | -0.13 [-0.30, 0.03] | 0.07 [-0.11, 0.25] | NA | NA | 0.12 [-0.13, 0.38] | -0.03 [-0.33, 0.28] |
| Adolescent Depression | **Mid vs Early** | 0.18 [0.04, 0.33]* | 0.11 [-0.05, 0.26] | -0.08 [-0.22, 0.06] | 0.06 [-0.08, 0.20] | NA | NA | 0.07 [-0.18, 0.32] | -0.04 [-0.29, 0.20] |
| Adolescent Depression | **Late vs Early** | 0.31 [0.11, 0.50]** | 0.14 [-0.09, 0.37] | -0.14 [-0.29, 0.00]† | 0.09 [-0.07, 0.25] | NA | NA | 0.16 [-0.10, 0.41] | 0.00 [-0.28, 0.29] |
| Adolescent Life satisfaction | **Mid vs Early** | -0.28 [-0.44, -0.11]** | -0.13 [-0.32, 0.05] | 0.07 [-0.06, 0.20] | -0.02 [-0.16, 0.11] | NA | NA | -0.07 [-0.31, 0.18] | -0.04 [-0.25, 0.18] |
| Adolescent Life satisfaction | **Late vs Early** | -0.59 [-0.82, -0.36]*** | -0.28 [-0.56, -0.00] | 0.14 [0.01, 0.28]† | -0.00 [-0.16, 0.15] | NA | NA | -0.22 [-0.50, 0.06] | -0.15 [-0.43, 0.12] |

**Table S9**. Beta coefficients [and 95% CIs] for age at autism diagnosis on wellbeing outcomes for each family member for our sensitivity analyses using age at diagnosis as a categorical variable reflecting earlier (≤ 8 years), middle (9-14 years) and later (> 14 years) diagnosed. Asterisks and crosses indicate p-values after Benjamini-Hochberg FDR-adjustment: *** p < .001, ** p < .01, * p < .05, † p < .1.

|  | Child | | Father | | | Mother | |
| --- | --- | --- | --- | --- | --- | --- | --- |
| Outcome | **Unadjusted** | **Adjusted** | **Unadjusted** | **Adjusted** | | **Unadjusted** | **Adjusted** |
| Childhood Anxiety | -0.13 [-0.19, -0.07]*** | -0.11 [-0.19, -0.04]** | -0.04 [-0.09, 0.02] | -0.00 [-0.07, 0.07] | -0.12 [-0.17, -0.06]*** | | -0.01 [-0.08, 0.05] |
| Childhood Depression | -0.18 [-0.24, -0.12]*** | -0.08 [-0.15, -0.00]† | -0.04 [-0.09, 0.02] | 0.00 [-0.06, 0.07] | -0.16 [-0.22, -0.11]*** | | -0.04 [-0.12, 0.03] |
| Childhood Life satisfaction | 0.12 [0.06, 0.19]*** | 0.05 [-0.03, 0.12] | 0.04 [-0.01, 0.10] | -0.01 [-0.07, 0.05] | 0.17 [0.12, 0.23]*** | | 0.06 [-0.02, 0.13] |
| Adolescent Anxiety | 0.12 [0.06, 0.19]*** | 0.06 [-0.02, 0.14] | NA | NA | -0.06 [-0.12, 0.00]† | | 0.02 [-0.06, 0.10] |
| Adolescent Depression | 0.13 [0.05, 0.20]** | 0.06 [-0.04, 0.16] | NA | NA | -0.06 [-0.12, -0.01]† | | 0.03 [-0.04, 0.09] |
| Adolescent Life satisfaction | -0.24 [-0.33, -0.15]*** | -0.12 [-0.24, 0.00] | NA | NA | 0.06 [0.00, 0.11]† | | -0.00 [-0.07, 0.06] |

**Table S10**. Beta coefficients [and 95% CIs] for age at autism diagnosis on wellbeing outcomes for each family member for our sensitivity analyses where we restricted the number of autistic children from each family to one. Asterisks and crosses indicate p-values after Benjamini-Hochberg FDR-adjustment: *** p < .001, ** p < .01, * p < .05, † p < .1.

**Figure S1**. Standardised regression coefficients for the remaining hypothesised confounders on age at first autism diagnosis. Light blue markers show coefficient estimates from simple regression models for each confounder, whereas dark blue markers show estimates from a multiple regression model.

**Figure S2.** Standardised regression coefficients for central clinical and sociodemographic factors, and children’s genetic liabilities on anxiety (blue), depression (green), and life satisfaction (yellow) of children during childhood.

**Figure S3.** Standardised regression coefficients for central clinical and sociodemographic factors, and children’s genetic liabilities on anxiety (blue), depression (green), and life satisfaction (yellow) of mothers when the child was 8 years old.

**Figure S4.** Standardised regression coefficients for central clinical and sociodemographic factors, and children’s genetic liabilities on anxiety (blue), depression (green), and life satisfaction (yellow) of children during adolescence.

**Figure S5.** Standardised regression coefficients for central clinical and sociodemographic factors, and children’s genetic liabilities on anxiety (blue), depression (green), and life satisfaction (yellow) of mothers when the child was 14 years old.

**Figure S6**. Associations between age at first autism diagnosis and measures of paternal wellbeing when the autistic children were 8 years old. The bold light blue lines represent the regression slopes for the unadjusted models, whereas the bold dark blue lines represent the regression slopes for the adjusted models. The light and dark ribbons represent the respective 95% confidence intervals.

**Figure S7**. Associations between age at first autism diagnosis and measures of sibling wellbeing when the autistic children were 8 years old. The bold light blue lines represent the regression slopes for the unadjusted models, whereas the bold dark blue lines represent the regression slopes for the adjusted models. The light and dark ribbons represent the respective 95% confidence intervals.

**Figure S8**. Associations between age at first autism diagnosis and measures of child wellbeing when the autistic children were 14 years old. The bold light blue lines represent the regression slopes for the unadjusted models, whereas the bold dark blue lines represent the regression slopes for the adjusted models. The light and dark ribbons represent the respective 95% confidence intervals.

**Figure S9**. Associations between age at first autism diagnosis and measures of maternal wellbeing when the autistic children were 14 years old. The bold light blue lines represent the regression slopes for the unadjusted models, whereas the bold dark blue lines represent the regression slopes for the adjusted models. The light and dark ribbons represent the respective 95% confidence intervals.

**Figure S10**. Associations between age at first autism diagnosis and measures of sibling wellbeing when the autistic children were 14 years old. The bold light blue lines represent the regression slopes for the unadjusted models, whereas the bold dark blue lines represent the regression slopes for the adjusted models. The light and dark ribbons represent the respective 95% confidence intervals.

**Figure S11.** Comparison of the associations between age at first autism and measures of paternal wellbeing when the autistic children were 8 years old by sex. The results are from the adjusted model controlling for all hypothesised confounders. The bold blue lines represent the regression slopes for boys, whereas the bold red lines represent the regression slopes for girls. The blue and red ribbons represent the respective 95% confidence intervals.

**Figure S12.** Comparison of the associations between age at first autism and measures of sibling wellbeing when the autistic children were 8 years old by sex. The results are from the adjusted model controlling for all hypothesised confounders. The bold blue lines represent the regression slopes for boys, whereas the bold red lines represent the regression slopes for girls. The blue and red ribbons represent the respective 95% confidence intervals.

**Figure S13.** Comparison of the associations between age at first autism and measures of the autistic children’s wellbeing when the autistic children were 14 years old by sex. The results are from the adjusted model controlling for all hypothesised confounders. The bold blue lines represent the regression slopes for boys, whereas the bold red lines represent the regression slopes for girls. The blue and red ribbons represent the respective 95% confidence intervals.

**Figure S14.** Comparison of the associations between age at first autism and measures of maternal wellbeing when the autistic children were 14 years old by sex. The results are from the adjusted model controlling for all hypothesised confounders. The bold blue lines represent the regression slopes for boys, whereas the bold red lines represent the regression slopes for girls. The blue and red ribbons represent the respective 95% confidence intervals.

**Figure S15.** Comparison of the associations between age at first autism and measures of sibling wellbeing when the autistic children were 14 years old by sex. The results are from the adjusted model controlling for all hypothesised confounders. The bold blue lines represent the regression slopes for boys, whereas the bold red lines represent the regression slopes for girls. The blue and red ribbons represent the respective 95% confidence intervals.

References

Angold, A., Costello, E., Messer, S., Pickles, A., Winder, F., & Silver, D. (1995). The Development of a Questionnaire for Use in Epidemiological Studies of Depression in Children and Adolescents. *International Journal of Methods in Psychiatric Research*, *5*, 237–249.

Birmaher, B., Brent, D. A., Chiappetta, L., Bridge, J., Monga, S., & Baugher, M. (1999). Psychometric Properties of the Screen for Child Anxiety Related Emotional Disorders (SCARED): A Replication Study. *Journal of the American Academy of Child & Adolescent Psychiatry*, *38*(10), 1230–1236. https://doi.org/10.1097/00004583-199910000-00011

Derogatis, L. R., Lipman, R. S., Covi, L., & others. (1973). SCL-90: An outpatient psychiatric rating scale–preliminary report. *Psychopharmacol Bull*, *9*(1), 13–28.

Diener, E., Emmons, R. A., Larsen, R. J., & Griffin, S. (1985). The Satisfaction With Life Scale. *Journal of Personality Assessment*, *49*(1), 71–75. https://doi.org/10.1207/s15327752jpa4901_13

Little, R. J. A. (1988). A Test of Missing Completely at Random for Multivariate Data with Missing Values. *Journal of the American Statistical Association*, *83*(404), 1198–1202. https://doi.org/10.1080/01621459.1988.10478722

Mervielde, I., & De Fruyt, F. (1999). Construction of the Hierarchical Personality Inventory for Children (HiPIC). *Personality Psychology in Europe. Proceedings of the Eight European Conference on Personality Psychology / I. Mervielde, I. Deary, F. De Fruyt, & F. Ostendorf (Eds.). - Tilburg : Tilburg University Press, 1999*, 107–127.

Mervielde, I., & De Fruyt, F. (2002). Assessing children’s traits with the hierarchial personality inventory for children. In *Big five assessment* (pp. 129–142). Hogrefe & Huber Publishers.

Privé, F., Albiñana, C., Arbel, J., Pasaniuc, B., & Vilhjálmsson, B. J. (2023). Inferring disease architecture and predictive ability with LDpred2-auto. *The American Journal of Human Genetics*, *110*(12), 2042–2055. https://doi.org/10.1016/j.ajhg.2023.10.010

Privé, F., Arbel, J., & Vilhjálmsson, B. J. (2020). LDpred2: Better, faster, stronger. *Bioinformatics*, *36*(22–23), 5424–5431. https://doi.org/10.1093/bioinformatics/btaa1029

Robins, D. L., Fein, D., Barton, M. L., & Green, J. A. (2001). The Modified Checklist for Autism in Toddlers: An Initial Study Investigating the Early Detection of Autism and Pervasive Developmental Disorders. *Journal of Autism and Developmental Disorders*, *31*(2), 131–144. https://doi.org/10.1023/A:1010738829569

Rubin, D. B. (1987). *Multiple Imputation for Nonresponse in Surveys*. John Wiley & Sons. http://dx.doi.org/10.1002/9780470316696

Stoltenberg, C., Schjølberg, S., Bresnahan, M., Hornig, M., Hirtz, D., Dahl, C., Lie, K. K., Reichborn-Kjennerud, T., Schreuder, P., Alsaker, E., Øyen, A.-S., Magnus, P., Surén, P., Susser, E., & Lipkin, W. I. (2010). The Autism Birth Cohort: A paradigm for gene–environment–timing research. *Molecular Psychiatry*, *15*(7), 676–680. https://doi.org/10.1038/mp.2009.143

Swinkels, S. H. N., Dietz, C., van Daalen, E., Kerkhof, I. H. G. M., van Engeland, H., & Buitelaar, J. K. (2006). Screening for Autistic Spectrum in Children Aged 14 to 15 Months. I: The Development of the Early Screening of Autistic Traits Questionnaire (ESAT). *Journal of Autism and Developmental Disorders*, *36*(6), 723–732. https://doi.org/10.1007/s10803-006-0115-0

Tambs, K., & Røysamb, E. (2014). Selection of questions to short-form versions of original psychometric instruments in MoBa. *Norsk Epidemiologi*, *24*(1–2). https://doi.org/10.5324/nje.v24i1-2.1822
